# Is a Mask That Covers the Mouth and Nose Free from Undesirable Side Effects in Everyday Use and Free of Potential Hazards?

**DOI:** 10.3390/ijerph18084344

**Published:** 2021-04-20

**Authors:** Kai Kisielinski, Paul Giboni, Andreas Prescher, Bernd Klosterhalfen, David Graessel, Stefan Funken, Oliver Kempski, Oliver Hirsch

**Affiliations:** 1 Private Practice, 40212 Düsseldorf, Germany; kaikisielinski@yahoo.de; 2 Private Practice, 22763 Hamburg, Germany; pgiboni@gmx.de; 3 Institute of Molecular and Cellular Anatomy (MOCA), Wendlingweg 2, 52074 Aachen, Germany; aprescher@ukaachen.de; 4 Institute of Pathology, Dueren Hospital, Roonstrasse 30, 52351 Dueren, Germany; bernd.klosterhalfen@web.de; 5 Institute of Neuroscience and Medicine, Forschungszentrum Jülich, 52425 Jülich, Germany; d.graessel@fz-juelich.de; 6 Private Practice, 47803 Krefeld, Germany; dr_funken@colita.net; 7 Institute of Neurosurgical Pathophysiology, University Medical Centre of the Johannes Gutenberg University of Mainz Langenbeckstr. 1, 55131 Mainz, Germany; oliver.kempski@unimedizin-mainz.de; 8 Department of Psychology, FOM University of Applied Sciences, 57078 Siegen, Germany

**Keywords:** personal protective equipment, masks, N95 face mask, surgical mask, risk, adverse effects, long-term adverse effects, contraindications, health risk assessment, hypercapnia, hypoxia, headache, dyspnea, physical exertion, MIES syndrome

## Abstract

Many countries introduced the requirement to wear masks in public spaces for containing SARS-CoV-2 making it commonplace in 2020. Up until now, there has been no comprehensive investigation as to the adverse health effects masks can cause. The aim was to find, test, evaluate and compile scientifically proven related side effects of wearing masks. For a quantitative evaluation, 44 mostly experimental studies were referenced, and for a substantive evaluation, 65 publications were found. The literature revealed relevant adverse effects of masks in numerous disciplines. In this paper, we refer to the psychological and physical deterioration as well as multiple symptoms described because of their consistent, recurrent and uniform presentation from different disciplines as a Mask-Induced Exhaustion Syndrome (MIES). We objectified evaluation evidenced changes in respiratory physiology of mask wearers with significant correlation of O_2_ drop and fatigue (*p* < 0.05), a clustered co-occurrence of respiratory impairment and O_2_ drop (67%), N95 mask and CO_2_ rise (82%), N95 mask and O_2_ drop (72%), N95 mask and headache (60%), respiratory impairment and temperature rise (88%), but also temperature rise and moisture (100%) under the masks. Extended mask-wearing by the general population could lead to relevant effects and consequences in many medical fields.

## 1. Introduction

At the beginning of the spread of the novel pathogen SARS-CoV-2, it was necessary to make far-reaching decisions even without available explicit scientific data. The initial assumption was that the pandemic emergency measures were set in place to reduce the acute threat of the public health system effectively and swiftly.

In April 2020, the World Health Organization (WHO) recommended the use of masks only for symptomatic, ill individuals and health care workers and did not recommend its widespread use.

In June 2020, they changed this recommendation to endorse the general use of masks in, e.g., crowded places [1,2]. In a meta-analysis study commissioned by the WHO (evidence level Ia), no clear, scientifically graspable benefit of moderate or strong evidence was derived from wearing masks [3].

While maintaining a distance of at least one meter showed moderate evidence with regard to the spreading of SARS-CoV-2, only weak evidence at best could be found for masks alone in everyday use (non-medical setting) [3]. Another meta-analysis conducted in the same year confirmed the weak scientific evidence for masks [4].

Accordingly, the WHO did not recommend general or uncritical use of masks for the general population and expanded its risk and hazard list within just two months. While the April 2020 guideline highlighted the dangers of self-contamination, possible breathing difficulties and false sense of security, the June 2020 guideline found additional potential adverse effects such as headache, development of facial skin lesions, irritant dermatitis, acne or increased risk of contamination in public spaces due to improper mask disposal [1,2].

However, under pressure from increasing absolute numbers of positive SARS-CoV-2 tests, many prescribers further extended mask-wearing according to certain times and situations, always justified by the desire to limit the spread of the virus [5]. The media, numerous institutions and most of the population supported this approach.

Among the medical profession and scientists, the users and observers of medical devices, there have been simultaneous calls for a more nuanced approach [6,7,8]. While there has been a controversial scientific discussion worldwide about the benefits and risks of masks in public spaces, they became the new social appearance in everyday life in many countries at the same time.

Although there seems to be a consensus among the decision makers who have introduced mandatory masks that medical exemptions are warranted, it is ultimately the responsibility of individual clinicians to weigh up when to recommend exemption from mandatory masks. Physicians are in a conflict of interest concerning this matter. On the one hand, doctors have a leading role in supporting the authorities in the fight against a pandemic. On the other hand, doctors must, in accordance with the medical ethos, protect the interests, welfare and rights of their patient’s third parties with the necessary care and in accordance with the recognized state of medical knowledge [9,10,11].

A careful risk–benefit analysis is becoming increasingly relevant for patients and their practitioners regarding the potential long-term effects of masks. The lack of knowledge of legal legitimacy on the one hand and of the medical scientific facts on the other is a reason for uncertainty among clinically active colleagues.

The aim of this paper is to provide a first, rapid, scientific presentation of the risks of general mandatory mask use by focusing on the possible adverse medical effects of masks, especially in certain diagnostic, patient and user groups.

## 2. Materials and Methods

The objective was to search for documented adverse effects and risks of different types of mouth–nose-covering masks. Of interest here were, on the one hand, readymade and self-manufactured fabric masks, including so-called community masks and, on the other hand medical, surgical and N95 masks (FFP2 masks).

Our approach of limiting the focus to negative effects seems surprising at first glance. However, such an approach helps toprovide us with more information. This methodology is in line with the strategy of Villalonga-Olives and Kawachi, who also conducted a review exclusively on the negative effects [12].

For an analysis of the literature, we defined the risk of mouth–nose protection as the description of symptoms or the negative effects of masks. Reviews and expert presentations from which no measurable values could be extracted, but which clearly present the research situation and describe negative effects, also fulfill this criterion.

Additionally, we defined the quantifiable, negative effect of masks as the presentation of a measured, statistically significant change in a physiological parameter in a pathological direction (*p* < 0.05), a statistically significant detection of symptoms (*p* < 0.05) or the occurrence of symptoms in at least 50% of those examined in a sample (*n* ≥ 50%).

**Figure 1:**
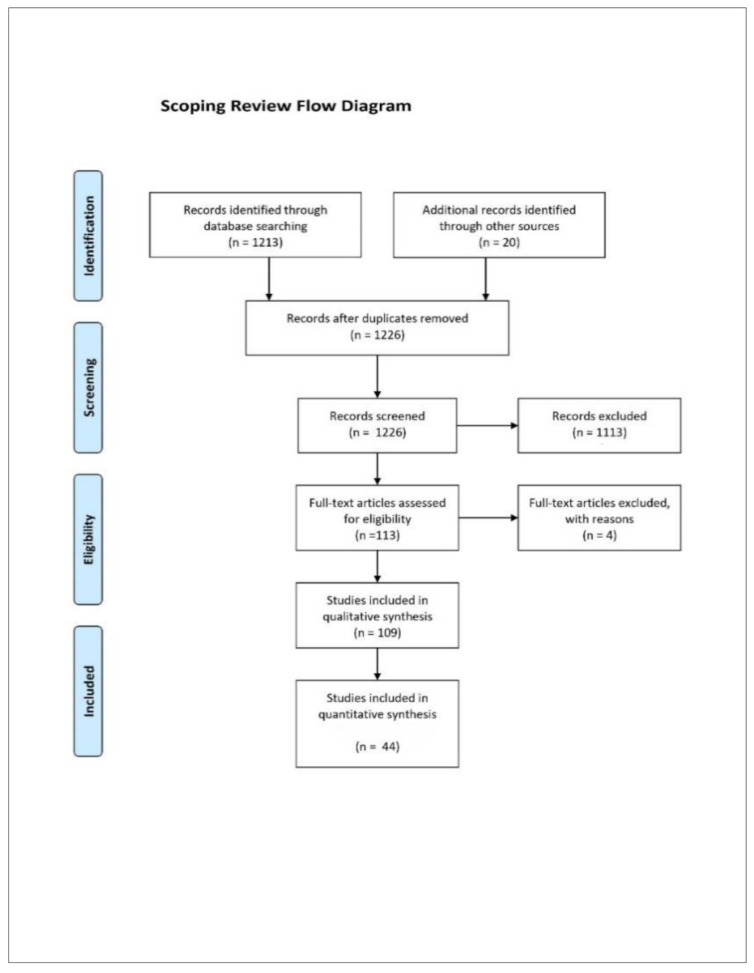
Scoping review flow diagram according to the PRISMA scheme.

Up to and including 31 October 2020, we conducted a database search in PubMed/MEDLINE on scientific studies and publications on adverse effects and risks of different types of mouth–nose-covering masks according to the criteria mentioned above (see Figure 1: Review flowchart). Terms searched were “face masks”, “surgical mask” and “N95” in combination with the terms “risk” and “adverse effects” as well as “side effects”. The selection criteria of the papers were based on our above definition of risk and adverse effect of masks. Mainly English- and German-language publications of evidence levels I to III according to the recommendations of the Agency for Healthcare Research and Quality (AHQR) that were not older than 20 years at the time of the review were considered. The evaluation also excluded level IV evidence, such as case reports and irrelevant letters to the editor that exclusively reflect opinions without scientific evidence.

After excluding 1113 papers that were irrelevant to the research question and did not meet the criteria mentioned (quantifiable, negative effects of masks, description of symptoms or the negative effects of masks), a total of 109 relevant publications were found for evaluation in the context of our scoping review (see Figure 1: Flow chart).

**Figure 2:**
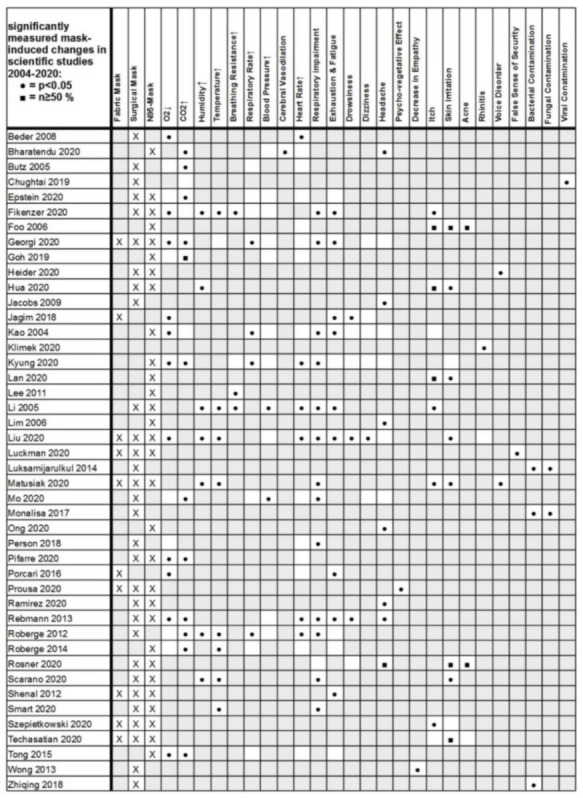
Overview including all 44 considered studies with quantified, significant adverse effects of masks (black dots and black rectangles). Not all studies examined each mentioned parameter, as focused or subject-related questions were often in the foreground. Gray fields correspond to a lack of coverage in the primary studies, white fields represent measured effects. We found an often combination of significant chemical, physical, physiological parameters and complaints. Drowsiness summarizes the symptom for any qualitative neurological deficits described in the scientific literature examined.

Sixty-five relevant publications concerning masks were considered being within the scope of the content-related evaluation. These included 14 reviews and 2 meta-analyses from the primary research. For the quantitative evaluation, 44 presentations of negative effects from the years 2004 to 2020 were eligible. Thirty-one of these studies were experimental (70%), and 13 studies were data collection studies in the sense of simple observational studies, especially in the dermatological field (30%). The observed study parameters and significant results from these 44 publications (*p* < 0.05 or *n* ≥ 50%) were compiled in an overall display (Figure 2). Based on this data, a correlation analysis of the observed mask effects was performed. This included a correlation calculation of the recorded symptoms and physiological changes (for nominally scaled, dichotomous variables according to Fisher using R, R Foundation for Statistical Computing, Vienna, Austria, version 4.0.2).

In addition, another 64 publications with a neighboring range of topics were consulted in connection with the mask effects we found. These included declarations, guidelines and legal principles. In order to expand the amount of data for the discussion, we proceeded according to the “snowball principle” by locating citations of selected papers in the bibliographies and including them where appropriate.

Since the findings from the topics presented for discussion were to an unexpected degree subject-related, we decided to divide the results according to the fields of medicine. Of course, there are overlaps between the respective fields, which we point out in detail.

## 3. Results

A total of 65 scientific papers on masks qualified for a purely content-based evaluation. These included 14 reviews and two meta-analyses.

Of the mathematically evaluable, groundbreaking 44 papers with significant negative mask effects (*p* < 0.05 or *n* ≥ 50%), 22 were published in 2020 (50%), and 22 were published before the COVID-19 pandemic. Of these 44 publications, 31 (70%) were of experimental nature, and the remainder were observational studies (30%). Most of the publications in question were English (98%). Thirty papers referred to surgical masks (68%), 30 publications related to N95 masks (68%), and only 10 studies pertained to fabric masks (23%).

Despite the differences between the primary studies, we were able to demonstrate a statistically significant correlation in the quantitative analysis between the negative side effects of blood-oxygen depletion and fatigue in mask wearers with *p* = 0.0454.

In addition, we found a mathematically grouped common appearance of statistically significant confirmed effects of masks in the primary studies (*p* < 0.05 and *n* ≥ 50%) as shown in Figure 2. In nine of the 11 scientific papers (82%), we found a combined onset of N95 respiratory protection and carbon dioxide rise when wearing a mask. We found a similar result for the decrease in oxygen saturation and respiratory impairment with synchronous evidence in six of the nine relevant studies (67%). N95 masks were associated with headaches in six of the 10 studies (60%). For oxygen deprivation under N95 respiratory protectors, we found a common occurrence in eight of 11 primary studies (72%). Skin temperature rise under masks was associated with fatigue in 50% (three out of six primary studies). The dual occurrence of the physical parameter temperature rise and respiratory impairment was found in seven of the eight studies (88%). A combined occurrence of the physical parameters temperature rise and humidity/moisture under the mask was found in 100% within six of six studies, with significant readings of these parameters (Figure 2).

The literature review confirms that relevant, undesired medical, organ and organ system-related phenomena accompanied by wearing masks occur in the fields of internal medicine (at least 11 publications, Section 3.2). The list covers neurology (seven publications, Section 3.3), psychology (more than 10 publications, Section 3.4), psychiatry (three publications, Section 3.5), gynecology (three publications, Section 3.6), dermatology (at least 10 publications, Section 3.7), ENT medicine (four publications, Section 3.8), dentistry (one publication, Section 3.8), sports medicine (four publications, Section 3.9), sociology (more than five publications, Section 3.10), occupational medicine (more than 14 publications, Section 3.11), microbiology (at least four publications, Section 3.12), epidemiology (more than 16 publications, Section 3.13), and pediatrics (four publications, Section 3.14) as well as environmental medicine (four publications, Section 3.15).

We will present the general physiological effects as a basis for all disciplines. This will be followed by a description of the results from the different medical fields of expertise and closing off with pediatrics the final paragraph.

### 3.1. General Physiological and Pathophysiological Effects for the Wearer

As early as 2005, an experimental dissertation (randomized crossover study) demonstrated that wearing surgical masks in healthy medical personnel (15 subjects, 18–40 years old) leads to measurable physical effects with elevated transcutaneous carbon dioxide values after 30 min [13]. The role of dead space volume and CO_2_ retention as a cause of the significant change (*p* < 0.05) in blood gases on the way to hypercapnia, which was still within the limits, was discussed in this article. Masks expand the natural dead space (nose, throat, trachea, bronchi) outwards and beyond the mouth and nose.

An experimental increase in the dead space volume during breathing increases carbon dioxide (CO_2_) retention at rest and under exertion and correspondingly the carbon dioxide partial pressure pCO_2_ in the blood (*p* < 0.05) [14].

As well as addressing the increased rebreathing of carbon dioxide (CO_2_) due to the dead space, scientists also debate the influence of the increased breathing resistance when using masks [15,16,17].

According to the scientific data, mask wearers as a whole show a striking frequency of typical, measurable, physiological changes associated with masks.

In a recent intervention study conducted on eight subjects, measurements of the gas content for oxygen (measured in O_2_ Vol%) and carbon dioxide (measured in CO_2_ ppm) in the air under a mask showed a lower oxygen availability even at rest than without a mask. A Multi-Rae gas analyzer was used for the measurements (RaeSystems^®^) (Sunnyvale, California CA, United States). At the time of the study, the device was the most advanced portable multivariant real-time gas analyzer. It is also used in rescue medicine and operational emergencies. The absolute concentration of oxygen (O_2_ Vol%) in the air under the masks was significantly lower (minus 12.4 Vol% O_2_ in absolute terms, statistically significant with *p* < 0.001) at 18.3% compared to 20.9% room air concentration. Simultaneously, a health-critical value of carbon dioxide concentration (CO_2_ Vol%) increased by a factor of 30 compared to normal room air was measured (ppm with mask versus 464 ppm without mask, statistically significant with *p* < 0.001) [18].

These phenomena are responsible for a statistically significant increase in carbon dioxide (CO_2_) blood content in mask wearers [19,20], on the one hand, measured transcutaneously via an increased PtcCO_2_ value [15,17,19,21,22], on the other hand, via end-expiratory partial pressure of carbon dioxide (PETCO_2_) [23,24] or, respectively, the arterial partial pressure of carbon dioxide (PaCO_2_) [25].

In addition to the increase in the wearer’s blood carbon dioxide (CO_2_) levels (*p* < 0.05) [13,15,17,19,21,22,23,24,25,26,27,28], another consequence of masks that has often been experimentally proven is a statistically significant drop in blood oxygen saturation (SpO_2_) (*p* < 0.05) [18,19,21,23,29,30,31,32,33,34]. A drop in blood oxygen partial pressure (PaO_2_) with the effect of an accompanying increase in heart rate (*p* < 0.05) [15,23,29,30,34] as well as an increase in respiratory rate (*p* < 0.05) [15,21,23,35,36] have been proven.

A statistically significant measurable increase in pulse rate (*p* < 0.05) and decrease in oxygen saturation SpO_2_ after the first (*p* < 0.01) and second hour (*p* < 0.0001) under a disposable mask (surgical mask) were reported by researchers in a mask intervention study they conducted on 53 employed neurosurgeons [30].

In another experimental study (comparative study), surgical and N95 masks caused a significant increase in heart rate (*p* < 0.01) as well as a corresponding feeling of exhaustion (*p* < 0.05). These symptoms were accompanied by a sensation of heat (*p* < 0.0001) and itching (*p* < 0.01) due to moisture penetration of the masks (*p* < 0.0001) in 10 healthy volunteers of both sexes after only 90 min of physical activity [35]. Moisture penetration was determined via sensors by evaluating logs (SCXI-1461, National Instruments, Austin, TX, USA).

These phenomena were reproduced in another experiment on 20 healthy subjects wearing surgical masks. The masked subjects showed statistically significant increases in heart rate (*p* < 0.001) and respiratory rate (*p* < 0.02) accompanied by a significant measurable increase in transcutaneous carbon dioxide PtcCO_2_ (*p* < 0.0006). They also complained of breathing difficulties during the exercise [15].

The increased rebreathing of carbon dioxide (CO_2_) from the enlarged dead space volume in mask wearers can reflectively trigger increased respiratory activity with increased muscular work as well as the resulting additional oxygen demand and oxygen consumption [17]. This is a reaction to pathological changes in the sense of an adaptation effect. A mask-induced drop in blood oxygen saturation value (SpO_2_) [30] or the blood oxygen partial pressure (PaO_2_) [34] can in turn additionally intensify subjective chest complaints [25,34].

The documented mask-induced changes in blood gases towards hypercapnia (in-creased carbon dioxide/CO_2_ blood levels) and hypoxia (decreased oxygen/O_2_ blood levels) may result in additional nonphysical effects such as confusion, decreased thinking ability and disorientation [23,36,37,38,39], including overall impaired cognitive abilities and decrease in psychomotoric abilities [19,32,38,39,40,41]. This highlights the importance of changes in blood gas parameters (O_2_ and CO_2_) as a cause of clinically relevant psychological and neurological effects. The above parameters and effects (oxygen saturation, carbon dioxide content, cognitive abilities) were measured in a study on saturation sensors (Semi-Tec AG, Therwil, Switzerland), using a Borg Rating Scale, Frank Scale, Roberge Respirator Comfort Scale and Roberge Subjective Symptoms-during-Work Scale, as well as with a Likert scale [19]. In the other main study, conventional ECG, capnography and symptom questionnaires were used in measuring carbon dioxide levels, pulse and cognitive abilities [23]. Other physiological data collection was done with pulse oximeters (Allegiance, MCGaw, USA), subjective complaints were assessed with a 5-point Likert scale and motoric speed was recorded with linear-position transducers (Tendo-Fitrodyne, Sport Machins, Trencin, Slovakia) [32]. Some researchers used standardized, anonymized questionnaires to collect data on subjective complaints associated with masks [37].

In an experimental setting with different mask types (community, surgical, N95) a significant increase in heart rate (*p* < 0.04), a decrease in oxygen saturation SpO_2_ (*p* < 0.05) with an increase in skin temperature under the mask (face) and difficulty of breathing (*p* < 0.002) were recorded in 12 healthy young subjects (students). In addition, the investigators observed dizziness (*p* < 0.03), listlessness (*p* < 0.05), impaired thinking (*p* < 0.03) and concentration problems (*p* < 0.02), which were also statistically significant when wearing masks [29].

According to other researchers and their publications, masks also interfere with temperature regulation, impair the field of vision and of non-verbal and verbal communication [15,17,19,36,37,42,43,44,45].

The above-mentioned measurable and qualitative physiological effects of masks can have implications in various areas of expertise in medicine.

It is known from pathology that not only supra-threshold stimuli exceeding normal limits have disease-relevant consequences. Subthreshold stimuli are also capable of causing pathological changes if the exposure time is long enough. Examples occur from the slightest air pollution by hydrogen sulfide resulting in respiratory problems (throat irritation, coughing, reduced absorption of oxygen) and neurological diseases (headaches, dizziness) [46]. Furthermore, subthreshold but prolonged exposure to nitrogen oxides and particulate matter is associated with an increased risk of asthma, hospitalization and higher overall mortality [47,48]. Low concentrations of pesticides are also associated with disease-relevant consequences for humans such as mutations, development of cancer and neurological disorders [49]. Likewise, the chronic subthreshold intake of arsenic is associated with an increased risk of cancer [50], subthreshold intake of cadmium with the promotion of heart failure [51], subthreshold intake of lead is associated with hypertension, renal metabolic disorders and cognitive impairment [52] or subthreshold intake of mercury with immune deficiency and neurological disorders [53]. Subliminal UV radiation exposure over long periods is also known to cause mutation-promoting carcinogenic effects (especially white skin cancer) [54].

The mask-induced adverse changes are relatively minor at first glance, but repeated exposure over longer periods in accordance with the above-mentioned pathogenetic principle is relevant. Long-term disease-relevant consequences of masks are to be expected. Insofar, the statistically significant results found in the studies with mathematically tangible differences between mask wearers and people without masks are clinically relevant. They give an indication that with correspondingly repeated and prolonged exposure to physical, chemical, biological, physiological and psychological conditions, some of which are subliminal, but which are significantly shifted towards pathological areas, health-reducing changes and clinical pictures can develop such as high blood pressure and arteriosclerosis, including coronary heart disease (metabolic syndrome) as well as neurological diseases. For small increases in carbon dioxide in the inhaled air, this disease-promoting effect has been proven with the creation of headaches, irritation of the respiratory tract up to asthma as well as an increase in blood pressure and heart rate with vascular damage and, finally, neuropathological and cardiovascular consequences [38]. Even slightly but persistently increased heart rates encourage oxidative stress with endothelial dysfunction, via increased inflammatory messengers, and finally, the stimulation of arteriosclerosis of the blood vessels has been proven [55]. A similar effect with the stimulation of high blood pressure, cardiac dysfunction and damage to blood vessels supplying the brain is suggested for slightly increased breathing rates over long periods [56,57]. Masks are responsible for the aforementioned physiological changes with rises in inhaled carbon dioxide [18,19,20,21,22,23,24,25,26,27,28], small sustained increases in heart rate [15,23,29,30,35] and mild but sustained increases in respiratory rates [15,21,23,34,36].

**Figure 3:**
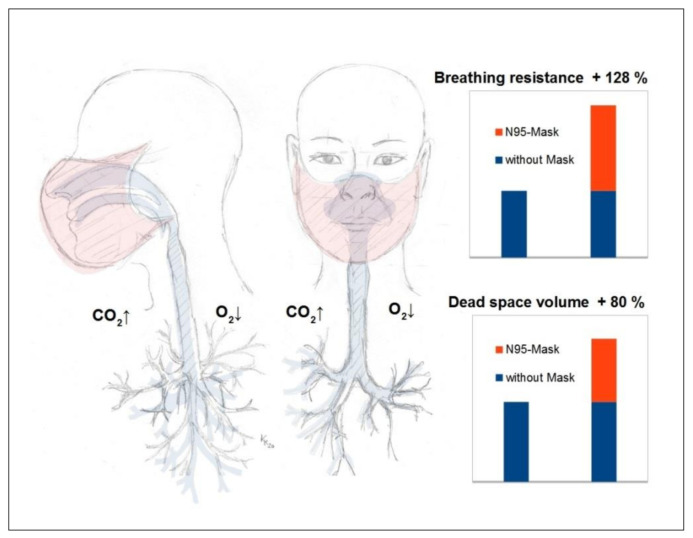
Pathophysiology of the mask (important physical and chemical effects): Illustration of the breathing resistance* and of the dead space volume of an N95 mask in an adult. When breathing, there is an overall significantly reduced possible gas exchange volume of the lungs of minus 37% caused by the mask (Lee 2011) [60] according to a decrease in breathing depth and volume due to the greater breathing resistance of plus128%* (exertion when inhaling greater than when exhaling) and due to the increased dead space volume of plus80%°, which does not participate directly in the gas exchange and is being only partially mixed with the environment. (* = averaged inspiration and expiration according to Lee 2011 [60] including moisture penetration according to Roberge 2010 [61], ** = averaged values according to Xu 2015 [59]).

For a better understanding of the side effects and dangers of masks presented in this literature review, it is possible to refer to well-known principles of respiratory physiology (Figure 3).

The average dead space volume during breathing in adults is approximately 150–180 mL and is significantly increased when wearing a mask covering the mouth and nose [58]. With an N95 mask, for example, the dead space volume of approximately 98–168 mL was determined in an experimental study [59]. This corresponds to a mask-related dead space increase of approximately 65 to 112% for adults and, thus, almost a doubling. At a respiratory rate of 12 per minute, the pendulum volume respiration with such a mask would, thus, be at least 2.9–3.8 L per minute. Therefore, the dead space amassed by the mask causes a relative reduction in the gas exchange volume available to the lungs per breath by 37% [60]. This largely explains the impairment of respiratory physiology reported in our work and the resulting side effects of all types of masks in everyday use in healthy and sick people (increase in respiratory rate, increase in heart rate, decrease in oxygen saturation, increase in carbon dioxide partial pressure, fatigue, headaches, dizziness, impaired thinking, etc.) [36,58].

In addition to the effect of increased dead space volume breathing, however, mask-related breathing resistance is also of exceptional importance (Figure 3) [23,36].

Experiments show an increase in airway resistance by a remarkable 126% on inhalation and 122% on exhalation with an N95 mask [60]. Experimental studies have also shown that moisturization of the mask (N95) increases the breathing resistance by a further 3% [61] and can, thus, increase the airway resistance up to 2.3 times the normal value.

This clearly shows the importance of the airway resistance of a mask. Here, the mask acts as a disturbance factor in breathing and makes the observed compensatory reactions with an increase in breathing frequency and simultaneous feeling of breathlessness plausible (increased work of the respiratory muscles). This extra strain due to the amplified work of breathing against bigger resistance caused by the masks also leads to intensified exhaustion with a rise in heart rate and increased CO_2_ production. Fittingly, in our review of the studies on side effects of masks (Figure 2), we also found a percentage clustering of significant respiratory impairment and a significant drop in oxygen saturation (in about 75% of all study results).

In the evaluation of the primary papers, we also determined a statically significant correlation of the drop in oxygen saturation (SpO_2_) and fatigue with a common occurrence in 58% of the mask use studies with significant results (Figure 2, *p* < 0.05).

### 3.2. Internistic Side Effects and Dangers

As early as 2012, an experiment showed that walking in the 20 masked subjects compared to the identical activity without masks significantly increased heart rates (average +9.4 beats per minute, *p* < 0.001) and breathing rates (*p* < 0.02). These physiological changes were accompanied by transcutaneous significantly measurable increased transcutaneous carbon dioxide (PtcCO_2_) levels (*p* < 0.0006) as well as respiratory difficulties in the mask wearers compared to the control group [15].

In a recent experimental comparative study from 2020, 12 healthy volunteers under surgical masks as well as under N95 masks experienced measurable impairments in the measured lung function parameters as well as cardiopulmonary capacity (lower maximum blood lactate response) during moderate to heavy physical exertion compared to exertion without masks (*p* < 0.001) [31]. The mask-induced increased airway resistance led to increased respiratory work with increased oxygen consumption and demand, both of the respiratory muscles and the heart. Breathing was significantly impeded (*p* < 0.001) and participants reported mild pain. The scientists concluded from their results that the cardiac compensation of the pulmonary, mask-induced restrictions, which still functioned in healthy people, was probably no longer possible in patients with reduced cardiac output [31].

In another recent study, researchers tested fabric masks (community masks), surgical masks and FFP2/N95 masks in 26 healthy people during exercise on a cycle ergometer. All masks also showed a measurable carbon dioxide (CO_2_) retention (PtcCO_2_) (statistically significant with *p* < 0.001) and, for N95 masks, a decrease in the oxygen saturation value SpO_2_ (statistically significant at 75 and 100 W with *p* < 0.02 and *p* < 0.005, respectively). The clinical relevance of these changes was shown in an increase in breathing frequency with fabric masks (*p* < 0.04) as well as in the occurrence of the previously described mask-specific complaints such as a feeling of heat, shortness of breath and headaches. The stress perception was recorded on a Borg scale from 1 to 20. During physical exertion under an N95 mask, the group with masks showed a significant increase in the feeling of exhaustion compared to the group without with 14.6 versus 11.9 on the scale of 20. During the exposure, 14 of the 24 subjects wearing masks complained of shortness of breath (58%), four of headaches and two of a feeling of heat. Most of the complaints concerned FFP2 masks (72%) [21].

The aforementioned physiological and subjective physical effects of masks on healthy people at rest and under exertion [21,31] give an indication of the effect of masks on sick and elderly people even without exertion.

In an observational study of ten 20 to 50 year-old nurses wearing N95 masks during their shift work, side effects such as breathing difficulties (“I can’t breathe”), feelings of exhaustion, headache (*p* < 0.001), drowsiness (*p* < 0.001) and a decrease in oxygen saturation SpO_2_ (*p* < 0.05) as well as an increase in heart rate (*p* < 0.001) were statistically significant in association with an increase in obesity (BMI) [19]. The occurrence of symptoms under masks was also associated with older age (statistically significant correlation of fatigue and drowsiness with *p* < 0.01 each, nausea with *p* < 0.05, an increase in blood pressure with *p* < 0.01, headache with *p* < 0.05, breathing difficulties with *p* < 0.001) [19].

In an intervention study involving 97 patients with advanced chronic obstructive pulmonary disease (COPD) the respiratory rate, oxygen saturation and exhaled carbon dioxide equivalents (capnometry) changed unfavorably and significantly after the use of N95 masks (FFP2 equivalent) with an initial 10-minute rest and subsequent 6-minute walking. Seven patients discontinued the experiment due to serious complaints with a decrease in the oxygen saturation value SpO_2_ and a pathological carbon dioxide (CO_2_) retention as well as increased end-expiratory partial pressure of carbon dioxide (PETCO_2_) [23]. In two patients, the PETCO_2_ exceeded the normal limits and reached values of >50 mmHg. An FEV1 < 30% and a modified Medical Research Council (mMRC) Dyspnea Scale Score of ≥3, both indicators of advanced COPD, correlated with mask intolerance overall in this study. The most common symptom under mask was breathlessness at 86%. In the dropouts of the study, dizziness (57%) and headaches were also often recorded. In the mask-tolerant COPD patients, significant increases in heart rate, respiratory rate and end-expiratory carbon dioxide partial pressure PETCO_2_ could be objectified even at rest, after only 10 min of mask-wearing (*p* < 0.001), accompanied by a decrease in oxygen saturation SpO_2_ (*p* < 0.001) [23]. The results of this study with an evidence level IIa are indicative for COPD mask wearers.

In another retrospective comparative study on COPD and surgical masks, examiners were able to demonstrate statistically an increase in arterial partial pressure of carbon dioxide (PaCO_2_) of approximately +8 mmHg (*p* < 0.005) and a concomitant mask-related increase in systolic blood pressure of +11 mmHg (*p* < 0.02) [25]. This increase is relevant in hypertensive patients, but also in healthy people with borderline blood pressure values as pathological value range triggered by mask-wearing can be induced.

In 39 hemodialysis patients with end-stage renal disease, a type N95 mask (FFP2 equivalent) caused a significant drop in blood oxygen partial pressure (PaO_2_) in 70% of patients at rest (on hemodialysis) within only 4 h (*p* = 0.006). Despite a compensatory increased respiratory rate (*p* < 0.001), malaise with chest pain occurred (*p* < 0.001) and even resulted in hypoxemia (drop in oxygen below the normal limit) in 19% of the subjects [34]. The researchers concluded from their findings that elderly or patients with reduced cardiopulmonary function have a higher risk of developing a severe respiratory failure while wearing a mask [34].

In a review paper on the risks and benefits of masks worn during the COVID-19 crisis, other authors provide an equally critical assessment of mandatory mask use for patients with pneumonia, both with and without COVID-19 pneumonia disease [16].

### 3.3. Neurological Side Effects and Dangers

In a scientific evaluation of syncope in the operating theatre, 36 of 77 affected persons (47%) were associated with wearing a mask [62]. However, other factors could not be ruled out as contributory causes.

In their level III evidence review, neurologists from Israel, the UK and the USA state that a mask is unsuitable for epileptics because it can trigger hyperventilation [63]. The use of a mask significantly increases the respiratory rate by about plus 15 to 20% [15,21,23,34,64]. However, an increase in breathing frequency leading to hyperventilation is known to be used for provocation in the diagnosis of epilepsy and causes seizure-equivalent EEG changes in 80% of patients with generalized epilepsy and in up to 28% of focal epileptics [65].

Physicians from New York studied the effects of wearing masks of the surgical-type mask and N95 among medical personnel in a sample of 343 participants (surveyed using standardized, anonymized questionnaires). Wearing the masks caused detectable physical adverse effects such as impaired cognition (24% of wearers) and headaches in 71.4% of the participants. Of these, 28% persisted and required medication. Headache occurred in 15.2% under 1 h of wear, in 30.6% after 1 h of wear and in 29.7% after 3 h of wear. Thus, the effect intensified with increasing wearing time [37].

Confusion, disorientation and even drowsiness (Likert scale questionnaire) and reduced motoric abilities (measured with a linear position transducer) with reduced reactivity and overall impaired performance (measured with the Roberge Subjective Symptoms-during-Work Scale) as a result of mask use have also been documented in other studies [19,23,29,32,36,37].

The scientists explain these neurological impairments with a mask-induced latent drop in blood gas oxygen levels O_2_ (towards hypoxia) or a latent increase in blood gas carbon dioxide levels CO_2_ (towards hypercapnia) [36]. In view of the scientific data, this connection also appears to be indisputable [38,39,40,41].

In a mask experiment from 2020, significant impaired thinking (*p* < 0.03) and impaired concentration (*p* < 0.02) were found for all mask types used (fabric, surgical and N95 masks) after only 100 min of wearing the mask [29]. The thought disorders correlated significantly with a drop in oxygen saturation (*p* < 0.001) during mask use.

Initial headaches (*p* < 0.05) were experienced by up to 82% of 158, 21–35 year-old mask wearers in another study of N95 respiratory protection with one third (34%) experiencing headaches up to four times daily. Participants wore the mask for 18.3 days over a 30-day period with a mean of 5.9 h per day [66].

Significantly increased headache (*p* < 0.05) could be observed not only for N95 but also for surgical masks in participants of another observational study of health care workers [67].

In another study, the researchers classified 306 users with an average age of 43 years and wearing different types of masks, of whom 51% had an initial headache as a specific symptom related exclusively to increased surgical and N95 mask use (1 to 4 h, *p* = 0.008) [68].

Researchers from Singapore were able to demonstrate in a trial involving 154 healthy N95 health service mask wearers that a significant increase in mask-induced blood carbon dioxide levels (measured by end-expiratory partial pressure of carbon dioxide PETCO_2_) and a measurably greater vasodilatation with an increase in cerebral artery flow in the cerebri media resulted. This was associated with headaches in the trial group (*p* < 0.001) [27].

According to the researchers, the aforementioned changes also contribute to headaches during the prolonged use of masks with a shift towards hypoxia and hypercapnia. Furthermore, stress and mechanical factors such as the irritation of cervical nerves in the neck and head area caused by the tight mask straps pressuring the nerve strands also contribute to headaches [66].

In the analysis of the primary studies, we were able to detect an association between the N95 mask and headaches. In six out of 10 studies, the significant headache appeared in conjunction with the N95 mask (60% of all studies, Figure 2).

### 3.4. Psychological Side Effects and Dangers

According to an experimental study, wearing surgical masks and N95 masks can also lead to a reduced quality of life owing to reduced cardiopulmonary capacity [31]. Masks, along with causing physiological changes and discomfort with progressive length of use, can also lead to significant discomfort (*p* < 0.03 to *p* < 0.0001) and a feeling of exhaustion (*p* < 0.05 to 0.0001) [69].

Besides the shift in blood gases towards hypercapnia (increase in CO_2_) and hypoxia (decrease in O_2_), detailed under general physiological effects (Section 3.1), masks also restrict the cognitive abilities of the individual (measured using a Likert scale survey) accompanied by a decline in psycho-motoric abilities and consequently a reduced responsiveness (measured using a linear position transducer) as well as an overall reduced performance capability (measured with the Roberge Subjective Symptoms-during-Work Scale) [29,32,38,39,41].

The mask also causes an impaired field of vision (especially affecting the ground and obstacles on the ground) and also presents an inhibition to habitual actions such as eating, drinking, touching, scratching and cleaning the otherwise uncovered part of the face, which is consciously and subconsciously perceived as a permanent disturbance, obstruction and restriction [36]. Wearing masks, thus, entails a feeling of deprivation of freedom and loss of autonomy and self-determination, which can lead to suppressed anger and subconscious constant distraction, especially as the wearing of masks is mostly dictated and ordered by others [70,71]. These perceived interferences of integrity, self-determination and autonomy, coupled with discomfort, often contribute to substantial distraction and may ultimately be combined with the physiologically mask-related decline in psycho-motoric abilities, reduced responsiveness and an overall impaired cognitive performance. It leads to misjudging situations as well as delayed, incorrect and inappropriate behavior and a decline in the effectiveness of the mask wearer [36,37,39,40,41].

The use of masks for several hours often causes further detectable adverse effects such as headaches, local acne, mask-associated skin irritation, itching, sensations of heat and dampness, impairments and discomfort predominantly affecting the head and face [19,29,35,36,37,71,72,73]. However, the head and face are significant for well-being due to their large representation in the sensitive cerebral cortex (homunculus) [36].

According to a questionnaire survey, masks also frequently cause anxiety and psycho-vegetative stress reactions in children—as well as in adults—with an increase in psychosomatic and stress-related illnesses and depressive self-experience, reduced participation, social withdrawal and lowered health-related self-care [74]. Over 50% of the mask wearers studied had at least mild depressive feelings [74]. Additional fear-inducing and often exaggerated media coverage can further intensify this. A recent retrospective analysis of the general media in the context of the 2014 Ebola epidemic showed a scientific truth content of only 38% of all publicly published information [75]. Researchers classified a total of 28% of the information as provocative and polarizing and 42% as exaggerating risks. In addition, 72% of the media content aimed to stir up health-related negative feelings. The feeling of fear, combined with insecurity and the primal human need to belong [76], causes a social dynamic that seems partly unfounded from a medical and scientific point of view.

The mask, which originally served purely hygienic purpose, has been transformed into a symbol of conformity and pseudo-solidarity. The WHO, for example, lists the advantages of the use of masks by healthy people in public to include a potentially reduced stigmatization of mask wearers, a sense of contribution to preventing the spread of the virus and a reminder to comply with other measures [2].

### 3.5. Psychiatric Side Effects and Dangers

As explained earlier, masks can cause increased rebreathing with an accumulation of carbon dioxide in the wearer due to increased dead space volume [16,17,18,20] (Figure 3), with often statistically significant measurable elevated blood carbon dioxide (CO2) levels in sufferers [13,15,17,19,20,21,22,23,24,25,26,27,28] (Figure 2). However, changes that lead to hypercapnia are known to trigger panic attacks [77,78]. This makes the significantly measurable increase in CO_2_ caused by wearing a mask clinically relevant.

Interestingly, breath provocation tests by inhaling CO_2_ are used to differentiate anxiety states in panic disorders and premenstrual dysphoria from other psychiatric clinical pictures. Here, absolute concentrations of 5% CO_2_ already suffice to trigger panic reactions within 15–16 min [77]. The normal exhaled air content of CO_2_ is about 4%.

It is obvious from experimental studies on masked subjects that concentration changes in the respiratory gases in the above-mentioned range with values above 4% could occur during rebreathing with prolonged mask use [18,23].

The activation of the locus coeruleus by CO_2_ is used to generate panic reactions via respiratory gases [78,79]. This is because the locus coeruleus is an important part of the system of vegetative noradrenergic neurons, a control center in the brainstem, which reacts to an appropriate stimulus and changes in the gas concentrations in the blood by releasing the stress hormone noradrenaline [78].

From the physiological, neurological and psychological side effects and dangers described above (Section 3.1, Section 3.3 and Section 3.4), additional problems can be derived for the use of masks in psychiatric cases. People undergoing treatment for dementia, paranoid schizophrenia, personality disorders with anxiety and panic attacks, but also panic disorders with claustrophobic components, are difficult to reconcile with a mask requirement, because even small increases in CO_2_ can cause and intensify panic attacks [44,77,78,79].

According to a psychiatric study, patients with moderate to severe dementia have no understanding of COVID-19 protection measures and have to be persuaded to wear masks constantly [80].

According to a comparative study, patients with schizophrenia have a lower acceptance of mask-wearing (54.9% agreement) than ordinary practice patients (61.6%) [81]. The extent to which mask-wearing can lead to an exacerbation of schizophrenia symptoms has not yet been researched in detail.

When wearing masks, confusion, impaired thinking, disorientation (standardized recording via special rating and Likert scales, *p* < 0.05) and in some cases a decrease in maximum speed and reaction time (measured with the linear-position transducer, *p* < 0.05) were observed [19,32,36,38,39,40,41]. Psychotropic drugs reduce psycho-motoric functions in psychiatric patients. This can become clinically relevant especially with regard to the further reduced ability to react and the additional increased susceptibility to accidents of such patients when wearing masks.

In order to avoid an unintentional CO_2_-triggered anesthesia [39], fixed and medically sedated patients, without the possibility of continuous monitoring, should not be masked according to the criteria of the Centers for Disease Control and Prevention, USA (CDC). This is because of the possible CO_2_ retention described above, as there is a risk of unconsciousness, aspiration and asphyxia [16,17,20,38,82,83].

### 3.6. Gynaecological Side Effects and Dangers

As a critical variable, a low blood carbon dioxide level in pregnant women is maintained via an increased respiratory minute volume, stimulated by progesterone [22]. For a pregnant woman and her unborn child, there is a metabolic need for a fetal–maternal carbon dioxide (CO_2_) gradient. The mother’s blood carbon dioxide level should always be lower than that of the unborn child in order to ensure the diffusion of CO_2_ from the fetal blood into the maternal circulation via the placenta.

Therefore, mask-related phenomena described above (Section 3.1 and Section 3.2), such as the measurable changes in respiratory physiology with increased breathing resistance, increased dead space volume (Figure 3) and the retention of exhaled carbon dioxide (CO_2_) are of importance. If CO_2_ is increasingly rebreathed under masks, this manifestation could, even with subliminal carbon dioxide increases, act as a disturbing variable of the fetal–maternal CO_2_ gradient increasing over time of exposure and, thus, develop clinical relevance, also with regard to a reduced compensation reserve of the expectant mothers [20,22,28].

In a comparative study, 22 pregnant women wearing N95 masks during 20 min of exercise showed significantly higher percutaneous CO_2_ values, with average PtcCO_2_ values of 33.3 mmHg compared to 31.3 mmHg than in 22 pregnant women without masks (*p* = 0.04) [22]. The heat sensation of the expectant mothers was also significantly increased with masks, with *p* < 0.001 [22].

Accordingly, in another intervention study, researchers demonstrated that breathing through an N95 mask (FFP2 equivalent) impeded gas exchange in 20 pregnant women at rest and during exercise, causing additional stress on their metabolic system [28]. Thus, under an N95 mask, 20 pregnant women showed a decrease in oxygen uptake capacity VO_2_ of about 14% (statistically significant, *p* = 0.013) and a decrease in carbon dioxide output capacity VCO_2_ of about 18% (statistically significant, *p* = 0.001). Corresponding significant changes in exhaled oxygen and carbon dioxide equivalents were also documented with increases in exhaled carbon dioxide (FeCO_2_) (*p* < 0.001) and decreases in exhaled oxygen (FeO_2_) (*p* < 0.001), which were explained by an altered metabolism due to respiratory mask obstruction [28].

In experiments with predominantly short mask application times, neither the mothers nor the fetuses showed statistically significant increases in heart rates or changes in respiratory rates and oxygen saturation values. However, the exact effects of prolonged mask use in pregnant women remain unclear overall. Therefore, in pregnant women, extended use of surgical and N95 masks is viewed critically [20].

In addition, it is unclear whether the substances contained in industrially manufactured masks that can be inhaled over longer periods of time (e.g., formaldehyde as an ingredient of the textile and thiram as an ingredient of the ear bands) are teratogenic [20,84].

### 3.7. Dermatological Side Effects and Dangers

Unlike garments worn over closed skin, masks cover body areas close to the mouth and nose, i.e., body parts that are involved with respiration.

Inevitably, this leads not only to a measurable temperature rise [15,44,85], but also to a severe increase in humidity due to condensation of the exhaled air, which in turn changes the natural skin milieu considerably of perioral and perinasal areas [36,61,82]. It also increases the redness, pH-value, fluid loss through the skin epithelium, increased hydration and sebum production measurably [73]. Preexisting skin diseases are not only perpetuated by these changes, but also exacerbated. In general, the skin becomes more susceptible to infections and acne.

The authors of an experimental study were able to prove a disturbed barrier function of the skin after only 4 h of wearing a mask in 20 healthy volunteers, both for surgical masks and for N95 masks [73]. In addition, germs (bacteria, fungi and viruses) accumulate on the outside and inside of the masks due to the warm and moist environment [86,87,88,89]. They can cause clinically relevant fungal, bacterial or viral infections. The unusual increase in the detection of rhinoviruses in the sentinel studies of the German Robert Koch Institute (RKI) from 2020 [90] could be another indication of this phenomenon.

In addition, a region of the skin that is not evolutionarily adapted to such stimuli is subjected to increased mechanical stress. All in all, the above-mentioned facts cause the unfavorable dermatological effects with mask related adverse skin reactions like acne, rashes on the face and itch symptoms [91].

A Chinese research group reported skin irritation and itching when using N95 masks among 542 test participants and also a correlation between the skin damage that occurred and the time of exposure (68.9% at ≤6 h/day and 81.7% at >6 h/day) [92].

A New York study evaluated in a random sample of 343 participants the effects of frequent wearing of surgical mask type and N95 masks among healthcare workers during the COVID-19 pandemic. Wearing the masks caused headache in 71.4% of participants, in addition to drowsiness in 23.6%, detectable skin damage in 51% and acne in 53% of mask users [37].

On the one hand, direct mechanical skin lesions occur on the nose and cheekbones due to shear force, especially when masks are frequently put on and taken off [37,92].

On the other hand, masks create an unnaturally moist and warm local skin environment [29,36,82]. In fact, scientists were able to demonstrate a significant increase in humidity and temperature in the covered facial area in another study in which the test individuals wore masks for one hour [85]. The relative humidity under the masks was measured with a sensor (Atmo-Tube, San Francisco, CA, USA). The sensation of humidity and temperature in the facial area is more crucial for well-being than other body regions [36,44]. This can increase discomfort under the masks. In addition, the increase in temperature favors bacterial optimization.

The pressure of the masks also causes an obstruction of the flow physiology of lymph and blood vessels in the face, with the consequence of increased disturbance of skin function [73] and ultimately also contributing to acne in up to 53% of all wearers and other skin irritations in up to 51% of all wearers [36,37,82].

Other researchers examined 322 participants with N95 masks in an observational study and detected acne in up to 59.6% of them, itching in 51.4% and redness in 35.8% as side effects [72].

In up to 19.6% (273) of the 1393 wearers of different masks (community masks, surgical, N95 masks), itching could be objectified in one study, in 9% even severely. An atopic predisposition (allergy tendency) correlated with the risk of itching. The length of use was significantly related to the risk of itching (*p* < 0.0001) [93].

In another dermatological study from 2020, 96.9% of 876 users of all mask types (community masks, surgical masks, N95 masks) confirmed adverse problems with a significant increase in itching (7.7%), accompanied by fogging-up of glasses (21.3%), flushing (21.3%), slurred speech (12.3%) and difficulty breathing (35.9%) (*p* < 0.01) [71].

Apart from an increased incidence of acne [37,72,91] under masks, contact eczema and urticaria [94] are generally described in connection with hypersensitivities to ingredients of the industrially manufactured masks (surgical mask and N95) such as formaldehyde (ingredient of the textile) and thiram (ingredient of the ear bands) [73,84]. The hazardous substance thiram, originally a pesticide and corrosive, is used in the rubber industry as a optimization accelerator. Formaldehyde is a biocide and carcinogen and is used as a disinfectant in the industry.

Even isolated permanent hyperpigmentation as a result of post-inflammatory or pigmented contact dermatitis has been described by dermatologists after prolonged mask use [72,91].

### 3.8. ENT and Dental Side Effects and Dangers

There are reports from dental communities about negative effects of masks and are accordingly titled “mask mouth” [95]. Provocation of gingivitis (inflammation of the gums), halitosis (bad breath), candidiasis (fungal infestation of the mucous membranes with Candida albicans) and cheilitis (inflammation of the lips), especially of the corners of the mouth, and even plaque and caries are attributed to the excessive and improper use of masks. The main trigger of the oral diseases mentioned is an increased dry mouth due to a reduced saliva flow and increased breathing through the open mouth under the mask. Mouth breathing causes surface dehydration and reduced salivary flow rate (SFR) [95]. Dry mouth is scientifically proven due to mask wear [29]. The bad habit of breathing through the open mouth while wearing a mask seems plausible because such breathing pattern compensates for the increased breathing resistance, especially when inhaling through the masks [60,61]. In turn, the outer skin moisture [71,73,85] with altered skin flora, which has already been described under dermatological side effects (Section 3.7), is held responsible as an explanation for the inflammation of the lips and corners of the mouth (cheilitis) [95]. This clearly shows the disease-promoting reversal of the natural conditions caused by masks. The physiological internal moisture with external dryness in the oral cavity converts into internal dryness with external moisture.

ENT physicians recently discovered a new form of irritant rhinitis due to N95 mask use in 46 patients. They performed endoscopies and nasal irrigations on mask wearers, which were subsequently assessed pathologically. Clinical problems were recorded with standardized questionnaires. They found statistically significant evidence of mask-induced rhinitis and itching and swelling of the mucous membranes as well as increased sneezing (*p* < 0.01). Endoscopically, it showed an increased secretion and evidence of inhaled mask polypropylene fibers as the trigger of mucosal irritation [96].

In a study of 221 health care workers, ENT physicians objectified a voice disorder in 33% of mask users. The VHI-10 score of 1 to 10, which measures voice disorders, was on average 5.72 higher in these mask users (statistically significant with *p* < 0.001). The mask not only acted as an acoustic filter, provoking excessively loud speech, it also seems to trigger impaired vocal cord coordination because the mask compromises the pressure gradients required for undisturbed speech [43]. The researchers concluded from their findings that masks could pose a potential risk of triggering new voice disorders as well as exacerbating existing ones.

### 3.9. Sports Medicine Side Effects and Dangers

According to the literature, performance-enhancing effects of masks regarding cardiovascular optimization and improvement of oxygen uptake capacity cannot be proven.

For example, in an experimental reference study (12 subjects per group), the training mask that supposedly mimics altitude training (ETM: elevation training mask) only had training effects on the respiratory muscles. However, mask wearers showed significantly lower oxygen saturation values (SpO_2_%) during exercise (SpO_2_ of 94% for mask wearers versus 96% for mask-less, *p* < 0.05) [33], which can be explained by an increased dead space volume and increased resistance during breathing. The measured oxygen saturation values were significantly lower than the normal values in the group of mask wearers, which indicates a clinical relevance.

The proven adaptation effect of the respiratory muscles in healthy athletes [33] clearly suggests that masks have a disruptive effect on respiratory physiology.

In another intervention study on mask use in weightlifters, researchers documented statistically significant effects of reduced attention (questionnaire recording, Likert scale) and a slowed maximum speed of movement detectable by means of sensors (both significant at *p* < 0.001), leading the researchers to conclude that mask use in sport is not without risks. As a secondary finding, they also detected a significant decrease in oxygen saturation SpO_2_ when performing special weight-lifting exercises (“back squats”) in the mask group after only 1 min of exercise compared to the mask-free group (*p* < 0.001) [32]. The proven tendency of the masks to shift the chemical parameter oxygen saturation SpO_2_ in a pathological direction (lower limit value 95%) may well have clinical relevance in untrained or sick individuals.

Sports medicine confirmed an increase in carbon dioxide (CO_2_) retention, with an elevation in CO_2_ partial pressure in the blood with larger respiratory dead space volumes [14].

In fact, dead space-induced CO_2_ retention while wearing a mask during exercise was also experimentally proven. The effects of a short aerobic exercise under N95 masks were tested on 16 healthy volunteers. A significantly increased end-expiratory partial pressure of carbon dioxide (PETCO_2_) with plus 8 mmHg (*p* < 0.001) was found [24]. The increase in blood carbon dioxide (CO_2_) in the mask wearers under maximum load was plus 14% CO_2_ for surgical masks and plus 23% CO_2_ for N95 masks, an effect that may well have clinical relevance in the pre-diseased, elderly and children, as these values strongly approached the pathological range [24].

In an interesting endurance study with eight middle-aged subjects (19–66), the gas content for O_2_ and CO_2_ under the masks was determined before and after exercise. Even at rest, the oxygen availability under the masks was 13% lower than without the masks and the carbon dioxide (CO_2_) concentration was 30 times higher. Under stress (Ruffier test), the oxygen concentration (% O_2_) below the mask dropped significantly by a further 3.7%, while the carbon dioxide concentration (% CO_2_) increased significantly by a further 20% (statistically significant with *p* < 0.001). Correspondingly, the oxygen saturation of the blood (SpO_2_) of the test persons also decreased significantly from 97.6 to 92.1% (*p* < 0.02) [18]. The drop in the oxygen saturation value (SpO_2_) to 92%, clearly below the normal limit of 95%, is to be classified as clinically relevant and detrimental to health.

These facts are an indication that the use of masks also triggers the effects described above leading to hypoxia and hypercapnia in sports. Accordingly, the WHO and Centers for Disease Control and Prevention, GA, USA (CDC) advise against wearing masks during physical exercise [82,97].

### 3.10. Social and Sociological Side Effects and Dangers

The results of a Chilean study with health care workers show that masks act like an acoustic filter and provoke excessively loud speech. This causes a voice disorder [43]. The increased volume of speech also contributes to increased aerosol production by the mask wearer [98]. These experimental data measured with the Aerodynamic Particle Sizer (APS, TSI, model 332, TSI Incorporated, Minnesota, MI, USA) are highly relevant.

Moreover, mask wearers are prevented from interacting normally in everyday life due to impaired clarity of speech [45], which tempts them to get closer to each other.

This results in a distorted prioritization in the general public, which counteracts the recommended measures associated with the COVID-19 pandemic. The WHO prioritizes social distancing and hand hygiene with moderate evidence and recommends wearing a mask with weak evidence, especially in situations where individuals are unable to maintain a physical distance of at least 1 m [3].

The disruption of non-verbal communication due to the loss of facial expression recognition under the mask can increase feelings of insecurity, discouragement and numbness as well as isolation, which can be extremely stressful for the mentally and hearing-impaired [16].

Experts point out that masks disrupt the basics of human communication (verbal and nonverbal). The limited facial recognition caused by masks leads to a suppression of emotional signals. Masks, therefore, disrupt social interaction, erasing the positive effect of smiles and laughter but at the same time greatly increasing the likelihood of misunderstandings because negative emotions are also less evident under masks [42].

A decrease in empathy perception through mask use with disruption of the doctor–patient relationship has already been scientifically proven on the basis of a randomized study (statistically significant, with *p* = 0.04) [99]. In this study, the Consultation Empathy Care Measury, the Patient Enablement Instrument (PEI) Score and a Satisfaction Rating Scale were assessed in 1030 patients. The 516 doctors, who wore masks throughout, conveyed reduced empathy towards the patients and, thus, nullified the positive health-promoting effects of a dynamic relationship. These results demonstrate a disruption of interpersonal interaction and relationship dynamics caused by masks.

The WHO guidance on the use of masks in children in the community, published in August 2020, points out that the benefits of mask use in children must be weighed up against the potential harms, including social and communicational concerns [100].

Fears that widespread pandemic measures will lead to dysfunctional social life with degraded social, cultural and psychological interactions have also been expressed by other experts [6,7,8,42].

### 3.11. Social and Occupational Medicine Side Effects and Hazards

In addition to mask-specific complaints such as a feeling of heat, dampness, shortness of breath and headache, various physiological phenomena were documented, such as the significant increase in heart and respiratory rate, the impairment of lung function parameters, the decrease in cardiopulmonary capacity (e.g., lower maximum blood lactate response) [15,19,21,23,29,30,31], as well as the changes in oxygen and carbon dioxide both in the end-expiratory and the air under the mask that was measured in the blood of the individuals [13,15,18,19,21,22,23,24,25,27,28,29,30,31,32,33,34]. The significant changes were measurable after only a few minutes of wearing a mask and in some cases reached magnitudes of minus 13% reduced O_2_ concentration and 30-fold increased CO_2_ concentration of the inhaled air under masks (*p* < 0.001) [18]. The changes observed were not only statistically significant, but also clinically relevant; the subjects also showed pathological oxygen saturation after exposure to masks (*p* < 0.02) [18].

Shortness of breath during light exertion (6 min walking) under surgical masks has been recorded with statistical significance in 44 healthy subjects in a prospective experimental intervention study (*p* < 0.001) [101]. Here, the complaints were assessed using a subjective, visual analogue scale.

In another study from 2011, all tested masks caused a significantly measurable increase in discomfort and a feeling of exhaustion in the 27 subjects during prolonged usage (*p* < 0.0001) [69].

These symptoms lead to additional stress for the occupational mask wearer and, thus, in relation to the feeling of exhaustion, contribute to the self-perpetuating vicious circle caused by the vegetative sympathetic activation, which further increases the respiratory and heart rate, blood pressure and increased sense of exhaustion [16,20,35,83].

Other studies showed that the psychological and physical effects of the masks can lead to an additional reduction in work performance (measured with the Roberge Subjective Symptoms-during-Work Scale, a Likert scale of 1–5) via increased feelings of fatigue, dissatisfaction and anxiety [58,102,103].

Wearing masks over a longer period of time also led to physiological and psychological impairments in other studies and, thus, reduced work performance [19,36,58,69]. In experiments on respiratory-protective equipment, an increase in the dead space volume by 350 mL leads to a reduction in the possible performance time by approx. −19%, furthermore to a decrease in breathing comfort by −18% (measured via a subjective rating scale) [58]. In addition, the time spent working and the flow of work is interrupted and reduced by putting on and taking off the masks and changing them. The reduced work performance has been recorded in the literature found as described above (especially in Section 3.1 and Section 3.2) but has not been quantified further in detail [36,58].

Surgical mask type and N95 protective equipment frequently caused adverse effects in medical personnel such as headaches, breathing difficulties, acne, skin irritation, itching, decreased alertness, decreased mental performance and feelings of dampness and heat [19,29,37,71,85]. Subjective, work performance-reducing, mask-related impairments in users, measured with special survey scores and Likert scales, have also been described in other studies [15,21,27,32,35,43,66,67,68,72,96,99].

In Section 3.7 on dermatology, we already mentioned a paper that demonstrated a significant temperature increase of 1.9 °C on average (to over 34.5 °C) in the mask-covered facial area (*p* < 0.05) [85]. Due to the relatively larger representation in the sensitive cerebral cortex (homunculus), the temperature sensation in the face is more decisive for the feeling of well-being than other body regions [36,44]. The perception of discomfort when wearing a mask can, thus, be intensified. Interestingly, in our analysis, we found a combined occurrence of the physical variable temperature rise under the mask and the symptom respiratory impairment in seven of eight studies concerned, with a mutual significantly measured occurrence in 88%. We also detected a combined occurrence of significantly measured temperature rise under the mask and significantly measured fatigue in 50% of the relevant primary studies (three of six papers, Figure 2). These clustered associations of temperature rise with symptoms of respiratory impairment and fatigue suggest a clinical relevance of the detected temperature rise under masks. In the worst case scenario, the effects mentioned can reinforce each other and lead to decompensation, especially in the presence of COPD, heart failure and respiratory insufficiency.

The sum of the disturbances and discomforts that can be caused by a mask also contributes to distraction (see also psychological impairment). These, in conjunction with a decrease in psycho-motoric skills, reduced responsiveness and overall impaired cognitive performance (all of which are pathophysiological effects of wearing a mask) [19,29,32,39,40,41] can lead to a failure to recognize hazards and, thus, to accidents or avoidable errors at work [19,36,37]. Of particular note here are mask-induced listlessness (*p* < 0.05), impaired thinking (*p* < 0.05) and concentration problems (*p* < 0.02) as measured by a Likert scale (1–5) [29]. Accordingly, occupational health regulations take action against such scenarios. The German Industrial Accident Insurance (DGUV) has precise and extensive regulations for respiratory protective equipment where they document the limitation of wearing time, levels of work intensity and defined instruction obligation [104].

The standards and norms prescribed in many countries regarding different types of masks to protect their workers are also significant from an occupational health point of view [105]. In Germany, for example, there are very strict safety specifications for masks from other international countries. These specify the requirements for the protection of the wearer [106]. All these standards and the accompanying certification procedures were increasingly relaxed with the introduction of mandatory masks for the general public. This meant that non-certified masks such as community masks were also used on a large scale in the work and school sectors for longer periods during the pandemic measures [107]. Most recently, in October 2020, the German Social Accident Insurance (DGUV) recommended the same usage time limits for community masks as for filtering half masks, namely, a maximum of three shifts of 120 min per day with recovery breaks of 30 min in between. In Germany, FFP2 (N95) masks must be worn for 75 min, followed by a 30-minute break. An additional suitability examination by specialized physicians is also obligatory and stipulated for occupationally used respirators [104].

### 3.12. Microbiological Consequences for Wearer and Environment: Foreign/Self-Contamination

Masks cause retention of moisture [61]. Poor filtration performance and incorrect use of surgical masks and community masks, as well as their frequent reuse, imply an increased risk of infection [108,109,110]. The warm and humid environment created by and in masks without the presence of protective mechanisms such as antibodies, the complement system, defense cells and pathogen-inhibiting and on a mucous membrane paves the way for unimpeded growth and, thus, an ideal growth and breeding ground for various pathogens such as bacteria and fungi [88] and also allows viruses to accumulate [87]. The warm and humid mask microclimate favors the accumulation of various germs on and underneath the masks [86], and the germ density is measurably proportional to the length of time the mask is worn. After only 2 h of wearing the mask, the pathogen density increases almost tenfold in experimental observation studies [87,89].

From a microbiological and epidemiological point of view, masks in everyday use pose a risk of contamination. This can occur as foreign contamination but also as self-contamination. On the one hand, germs are sucked in or attach themselves to the masks through convection currents. On the other hand, potential infectious agents from the nasopharynx accumulate excessively on both the outside and inside of the mask during breathing [5,88]. This is compounded by contact with contaminated hands. Since masks are constantly penetrated by germ-containing breath and the pathogen reproduction rate is higher outside mucous membranes, potential infectious pathogens accumulate excessively on the outside and inside of masks. On and in the masks, there are quite serious, potentially disease-causing bacteria and fungi such as *E. coli* (54% of all germs detected), Staphylococcus aureus (25% of all germs detected), Candida (6%), Klebsiella (5%), Enterococci (4%), Pseudomonads (3%), Enterobacter (2%) and Micrococcus (1%) even detectable in large quantities [88].

In another microbiological study, the bacterium Staphylococcus aureus (57% of all bacteria detected) and the fungus Aspergillus (31% of all fungi detected) were found to be the dominant germs on 230 surgical masks examined [86].

After more than six hours of use, the following viruses were found in descending order on 148 masks worn by medical personnel: adenovirus, bocavirus, respiratory syncytial virus and influenza viruses [87].

From this aspect, it is also problematic that moisture distributes these potential pathogens in the form of tiny droplets via capillary action on and in the mask, whereby further proliferation in the sense of self- and foreign contamination by the aerosols can then occur internally and externally with every breath [35]. In this regard, it is also known from the literature that masks are responsible for a proportionally disproportionate production of fine particles in the environment and, surprisingly, much more so than in people without masks [98].

It was shown that all mask-wearing subjects released significantly more smaller particles of size 0.3–0.5 μm into the air than mask-less people, both when breathing, speaking and coughing (fabric, surgical, N95 masks, measured with the Aerodynamic Particle Sizer, APS, TS, model 3329) [98]. The increase in the detection of rhinoviruses in the sentinel studies of the German RKI from 2020 [90] could be a further indication of this phenomenon, as masks were consistently used by the general population in public spaces in that year.

### 3.13. Epidemiological Consequences

The possible side effects and dangers of masks described in this paper are based on studies of different types of masks. These include the professional masks of the surgical mask type and N95/KN95 (FFP2 equivalent) that are commonly used in everyday life, but also the community fabric masks that were initially used. In the case of N95, the N stands for National Institute for Occupational Safety and Health of the United States (NIOSH), and 95 indicates the 95 per cent filtering capacity for fine particles up to at least 0.3 μm [82].

A major risk of mask use in the general public is the creation of a false sense of security with regard to protection against viral infections, especially in the sense of a falsely assumed strong self-protection. Disregarding infection risks may not only neglect aspects of source control, but also result in other disadvantages. Although there are quite a few professional positive accounts of the widespread use of masks in the general populace [111], most of the serious and evident scientific reports conclude that the general obligation to wear masks conveys a false sense of security [4,5]. However, this leads to a neglect of those measures that, according to the WHO, have a higher level of effectiveness than mask-wearing: social distancing and hand hygiene [2,112]. Researchers were able to provide statistically significant evidence of a false sense of security and more risky behavior when wearing masks in an experimental setting [112].

Decision makers in many countries informed their citizens early on in the pandemic in March 2020 that people without symptoms should not use a medical mask, as this created a false sense of security [113]. The recommendation was ultimately changed in many countries. At least Germany pointed out that wearers of certain types of masks such as the common fabric masks (community masks) cannot rely on them to protect them or others from transmission of SARS-CoV-2 [114].

However, scientists not only complain about the lack of evidence for fabric masks in the scope of a pandemic [16,110], but also about the high permeability of fabric masks with particles and the potential risk of infection they pose [108,109]. Ordinary fabric masks with a 97% penetration for particle dimensions of ≥0.3 μm are in stark contrast to medical-type surgical masks with a 44% penetration. In contrast, the N95 mask has a penetration rate of less than 0.01% for particles ≥ 0.3 μm in the laboratory experiment [108,115].

For the clinical setting in hospitals and outpatient clinics, the WHO guidelines recommend only surgical masks for influenza viruses for the entire patient treatment except for the strongly aerosol-generating measures, for which finer filtering masks of the type N95 are suggested. However, the WHO’s endorsement of specific mask types is not entirely evidence-based due to the lack of high-quality studies in the health sector [108,109,116,117].

In a laboratory experiment (evidence level IIa study), it was demonstrated that both surgical masks and N95 masks have deficits in protection against SARS-CoV-2 and influenza viruses using virus-free aerosols [118]. In this study, the FFP2-equivalent N95 mask performed significantly better in protection (8–12 times more effective) than the surgical mask, but neither mask type established reliable, hypothesis-generated protection against corona and influenza viruses. Both mask types could be penetrated unhindered by aerosol particles with a diameter of 0.08 to 0.2 μm. Both the SARS-CoV-2 pathogens with a size of 0.06 to 0.14 μm [119] and the influenza viruses with 0.08 to 0.12 μm are unfortunately well below the mask pore sizes [118].

The filtering capacity of the N95 mask up to 0.3 μm [82] is usually not achieved by surgical masks and community masks. However, aerosol droplets, which have a diameter of 0.09 to 3 μm in size, are supposed to serve as a transport medium for viruses. These also penetrate the medical masks by 40%. Often, there is also a poor fit between the face and the mask, which further impairs their function and safety [120]. The accumulation of aerosol droplets on the mask is problematic. Not only do they absorb nanoparticles such as viruses [6], but they also follow the airflow when inhaling and exhaling, causing them to be carried further. In addition, a physical decay process has been described for aerosol droplets at increasing temperatures, as also occurs under a mask [15,44,85]. This process can lead to a decrease in size of the fine water droplets up to the diameter of a virus [121,122]. The masks filter larger aerosol droplets but cannot retain viruses themselves and such smaller, potentially virus-containing aerosol droplets of less than 0.2 μm and hence cannot stop the spread of virus [123].

Similarly, in an in vivo comparative studies of N95 and surgical masks, there were no significant differences in influenza virus infection rates [124,125]. Although this contrasts with encouraging in vitro laboratory results with virus-free aerosols under non-natural conditions, even with fabric masks [126], it should be noted that under natural in-vivo conditions, the promising filtration functions of fabric masks based on electrostatic effects also rapidly diminish under increasing humidity [127]. A Swiss textile lab test of various masks available on the market to the general public recently confirmed that most mask types filter aerosols insufficiently. For all but one of the eight reusable fabric mask types tested, the filtration efficacy according to EN149 was always less than 70% for particles of 1 μm in size. For disposable masks, only half of all eight mask types tested were efficient enough at filtering to retain 70% of particles 1 μm in size [128].

A recent experimental study even demonstrated that all mask-wearing people (surgical, N95, fabric masks) release significantly and proportionately smaller particles of size 0.3 to 0.5 μm into the air than mask-less people, both when breathing, speaking and coughing [98]. According to this, the masks act like nebulizers and contribute to the production of very fine aerosols. Smaller particles, however, spread faster and further than large ones for physical reasons. Of particular interest in this experimental reference study was the finding that a test subject wearing a single-layer fabric mask was also able to release a total of 384% more particles (of various sizes) when breathing than a person without [98].

It is not only the aforementioned functional weaknesses of the masks themselves that lead to problems, but also their use. This increases the risk of a false sense of security. According to the literature, mistakes are made by both healthcare workers and lay people when using masks as hygienically correct mask use is by no means intuitive. Overall, 65% of healthcare professionals and as many as 78% of the general population, use masks incorrectly [116]. With both surgical masks and N95 masks, adherence to the rules of use is impaired and not adequately followed due to reduced wearability with heat discomfort and skin irritation [29,35,116,129]. This is exacerbated by the accumulation of carbon dioxide due to the dead space (especially under the N95 masks) with the resulting headaches described [19,27,37,66,67,68,83]. Increased heart rate, itching and feelings of dampness [15,29,30,35,71] also lead to reduced safety and quality during use (see also social and occupational health side effects and hazards). For this reason, (everyday) masks are even considered a general risk for infection in the general population, which does not come close to imitating the strict hygiene rules of hospitals and doctors’ offices: the supposed safety, thus, becomes a safety risk itself [5].

In a meta-analysis of evidence level Ia commissioned by the WHO, no effect of masks in the context of influenza virus pandemic prevention could be demonstrated [130]. In 14 randomized controlled trials, no reduction in the transmission of laboratory-confirmed influenza infections was shown. Due to the similar size and distribution pathways of the virus species (influenza and Corona, see above), the data can also be transferred to SARS-CoV-2 [118]. Nevertheless, a combination of occasional mask-wearing with adequate hand-washing caused a slight reduction in infections for influenza in one study [131]. However, since no separation of hand hygiene and masks was achieved in this study, the protective effect can rather be attributed to hand hygiene in view of the aforementioned data [131].

A recently published large prospective Danish comparative study comparing mask wearers and non-mask wearers in terms of their infection rates with SARS-CoV2 could not demonstrate any statistically significant differences between the groups [132].

### 3.14. Paediatric Side Effects and Hazards

Children are particularly vulnerable and may be more likely to receive inappropriate treatment or additional harm. It can be assumed that the potential adverse mask effects described for adults are all the more valid for children (see Section 3.1 to Section 3.13: physiological internal, neurological, psychological, psychiatric, dermatological, ENT, dental, sociological, occupational and social medical, microbiological and epidemiological impairments and also Figure 2 and Figure 3).

Special attention must be paid to the respiration of children, which represents a critical and vulnerable physiological variable due to higher oxygen demand, increased hypoxia susceptibility of the CNS, lower respiratory reserve, smaller airways with a stronger increase in resistance when the lumen is narrowed. The diving reflex caused by stimulating the nose and upper lip can cause respiratory arrest to bradycardia in the event of oxygen deficiency.

The masks currently used for children are exclusively adult masks manufactured in smaller geometric dimensions and had neither been specially tested nor approved for this purpose [133].

In an experimental British research study, the masks frequently led to feelings of heat (*p* < 0.0001) and breathing problems (*p* < 0.03) in 100 school children between 8 and 11 years of age especially during physical exertion, which is why the protective equipment was taken off by 24% of the children during physical activity [133]. The exclusion criteria for this mask experiment were lung disease, cardiovascular impairment and claustrophobia [133].

Scientists from Singapore were able to demonstrate in their level Ib study published in the renowned journal “nature” that 106 children aged between 7 and 14 years who wore FFP2 masks for only 5 min showed an increase in the inspiratory and expiratory CO_2_ levels, indicating disturbed respiratory physiology [26].

However, a disturbed respiratory physiology in children can have long-term disease-relevant consequences. Slightly elevated CO_2_ levels are known to increase heart rate, blood pressure, headache, fatigue and concentration disorders [38].

Accordingly, the following conditions were listed as exclusion criteria for mask use [26]: any cardiopulmonary disease including but not limited to: asthma, bronchitis, cystic fibrosis, congenital heart disease, emphysema; any condition that may be aggravated by physical exertion, including but not limited to: exercise-induced asthma; lower respiratory tract infections (pneumonia, bronchitis within the last 2 weeks), anxiety disorders, diabetes, hypertension or epilepsy/attack disorder; any physical disability due to medical, orthopedic or neuromuscular disease; any acute upper respiratory illness or symptomatic rhinitis (nasal obstruction, runny nose or sneezing); any condition with deformity that affects the fit of the mask (e.g., increased facial hair, craniofacial deformities, etc.).

It is also important to emphasize the possible effects of masks in neurological diseases, as described earlier (Section 3.3).

Both masks and face shields caused fear in 46% of children (37 out of 80) in a scientific study. If children are given the choice of whether the doctor examining them should wear a mask they reject this in 49% of the cases. Along with their parents, the children prefer the practitioner to wear a face visor (statistically significant with *p* < 0.0001) [134].

A recent observational study of tens of thousands of mask-wearing children in Germany helped the investigators objectify complaints of headaches (53%), difficulty concentrating (50%), joylessness (49%), learning difficulties (38%) and fatigue in 37% of the 25,930 children evaluated. Of the children observed, 25% had new onset anxiety and even nightmares [135]. In children, the threat scenarios generated by the environment are further maintained via masks, in some cases, even further intensified, and in this way, existing stress is intensified (presence of subconscious fears) [16,35,136,137].

This can in turn lead to an increase in psychosomatic and stress-related illnesses [74,75]. For example, according to an evaluation, 60% of mask wearers showed stress levels of the highest grade 10 on a scale of 1 to a maximum of 10. Less than 10% of the mask wearers surveyed had a stress level lower than 8 out of a possible 10 [74].

As children are considered a special group, the WHO also issued a separate guideline on the use of masks in children in the community in August 2020, explicitly advising policy makers and national authorities, given the limited evidence, that the benefits of mask use in children must be weighed up against the potential harms associated with mask use. This includes feasibility and discomfort, as well as social and communication concerns [100].

According to experts, masks block the foundation of human communication and the exchange of emotions and not only hinder learning but deprive children of the positive effects of smiling, laughing and emotional mimicry [42]. The effectiveness of masks in children as a viral protection is controversial, and there is a lack of evidence for their widespread use in children; this is also addressed in more detail by the scientists of the German University of Bremen in their thesis paper 2.0 and 3.0 [138].

### 3.15. Effects on the Environment

According to WHO estimates of a demand of 89 million masks per month, their global production will continue to increase under the Corona pandemic [139]. Due to the composition of, e.g., disposable surgical masks with polymers such as polypropylene, polyurethane, polyacrylonitrile, polystyrene, polycarbonate, polyethylene and polyester [140], an increasing global challenge, also from an environmental point of view, can be expected, especially outside Europe, in the absence of recycling and disposal strategies [139]. The aforementioned single use polymers have been identified as a significant source of plastic and plastic particles for the pollution of all water cycles up to the marine environment [141].

A significant health hazard factor is contributed by mask waste in the form of microplastics after decomposition into the food chain. Likewise, contaminated macroscopic disposable mask waste—especially before microscopic decay—represents a widespread medium for microbes (protozoa, bacteria, viruses, fungi) in terms of invasive pathogens [86,87,88,89,142]. Proper disposal of bio-contaminated everyday mask material is insufficiently regulated even in western countries.

## 4. Discussion

The potential drastic and undesirable effects found in multidisciplinary areas illustrate the general scope of global decisions on masks in general public in the light of combating the pandemic. According to the literature found, there are clear, scientifically recorded adverse effects for the mask wearer, both on a psychological and on a social and physical level.

Neither higher level institutions such as the WHO or the European Centre for Disease Prevention and Control (ECDC) nor national ones, such as the Centers for Disease Control and Prevention, GA, USA (CDC) or the German RKI, substantiate with sound scientific data a positive effect of masks in the public (in terms of a reduced rate of spread of COVID-19 in the population) [2,4,5].

Contrary to the scientifically established standard of evidence-based medicine, national and international health authorities have issued their theoretical assessments on the masks in public places, even though the compulsory wearing of masks gives a deceptive feeling of safety [5,112,143].

From an infection epidemiological point of view, masks in everyday use offer the risk of self-contamination by the wearer from both inside and outside, including via contaminated hands [5,16,88]. In addition, masks are soaked by exhaled air, which potentially accumulates infectious agents from the nasopharynx and also from the ambient air on the outside and inside of the mask. In particular, serious infection-causing bacteria and fungi should be mentioned here [86,88,89], but also viruses [87]. The unusual increase in the detection of rhinoviruses in the sentinel studies of the German RKI from 2020 [90] could be an indication of this phenomenon. Clarification through further investigations would therefore be desirable.

Masks, when used by the general public, are considered by scientists to pose a risk of infection because the standardized hygiene rules of hospitals cannot be followed by the general public [5]. On top of that, mask wearers (surgical, N95, fabric masks) exhale relatively smaller particles (size 0.3 to 0.5 μm) than mask-less people and the louder speech under masks further amplifies this increased fine aerosol production by the mask wearer (nebulizer effect) [98].

The history of modern times shows that already in the influenza pandemics of 1918–1919, 1957–58, 1968, 2002, in SARS 2004–2005 as well as with the influenza in 2009, masks in everyday use could not achieve the hoped-for success in the fight against viral infection scenarios [67,144]. The experiences led to scientific studies describing as early as 2009 that masks do not show any significant effect with regard to viruses in an everyday scenario [129,145]. Even later, scientists and institutions rated the masks as unsuitable to protect the user safely from viral respiratory infections [137,146,147]. Even in hospital use, surgical masks lack strong evidence of protection against viruses [67].

Originally born out of the useful knowledge of protecting wounds from surgeons’ breath and predominantly bacterial droplet contamination [144,148,149], the mask has been visibly misused with largely incorrect popular everyday use, particularly in Asia in recent years [150]. Significantly, the sociologist Beck described the mask as a cosmetic of risk as early as 1992 [151]. Unfortunately, the mask is inherent in a vicious circle: strictly speaking, it only protects symbolically and at the same time represents the fear of infection. This phenomenon is reinforced by the collective fear mongering, which is constantly nurtured by main stream media [137].

Nowadays, the mask represents a kind of psychological support for the general population during the virus pandemic, promising them additional anxiety-reduced freedom of movement. The recommendation to use masks in the sense of “source control” not out of self-protection but out of “altruism” [152] is also very popular with the regulators as well as the population of many countries. The WHO’s recommendation of the mask in the current pandemic is not only a purely infectiological approach, but is also clear on the possible advantages for healthy people in the general public. In particular, a reduced potential stigmatization of mask wearers, the feeling of a contribution made to preventing the spread of the virus, as well as the reminder to adhere to other measures are mentioned [2].

It should not go unmentioned that very recent data suggest that the detection of SARS-CoV-2 infection does not seem to be directly related to popular mask use. The groups examined in a retrospective comparative study (infected with SARS-CoV-2 and not infected) did not differ in their habit of using masks: approximately 70% of the subjects in both groups always wore masks and another 14.4% of them frequently [143].

In a Danish prospective study on mask-wearing carried out on about 6000 participants and published in 2020, scientists found no statistically significant difference in the rates of SARS-CoV-2 infection when comparing the group of 3030 mask wearers with the 2994 mask-less participants in the study (*p* = 0.38) [132].

Indeed, in the case of viral infections, masks appear to be not only less effective than expected, but also not free of undesirable biological, chemical, physical and psychological side effects [67]. Accordingly, some experts claim that well-intentioned unprofessionalism can be quite dangerous [6].

The dermatological colleagues were the first to describe common adverse effects of mask-wearing in larger collectives. Simple, direct physical, chemical and biological effects of the masks with increases in temperature, humidity and mechanical irritation caused acne in up to 60% of wearers [37,71,72,73,85]. Other significantly documented consequences were eczema, skin damage and overall impaired skin barrier function [37,72,73].

These direct effects of mask use are an important pointer to further detrimental effects affecting other organ systems.

In our work, we have identified scientifically validated and numerous statistically significant adverse effects of masks in various fields of medicine, especially with regard to a disruptive influence on the highly complex process of breathing and negative effects on the respiratory physiology and gas metabolism of the body (see Figure 2 and Figure 3). The respiratory physiology and gas exchange play a key role in maintaining a health-sustaining balance in the human body [136,153]. According to the studies we found, a dead space volume that is almost doubled by wearing a mask and a more than doubled breathing resistance (Figure 3) [59,60,61] lead to a rebreathing of carbon dioxide with every breathing cycle [16,17,18,39,83] with—in healthy people mostly—a subthreshold but, in sick people, a partly pathological increase in the carbon dioxide partial pressure (PaCO_2_) in the blood [25,34,58]. According to the primary studies found, these changes contribute reflexively to an increase in respiratory frequency and depth [21,23,34,36] with a corresponding increase in the work of the respiratory muscles via physiological feedback mechanisms [31,36]. Thus, it is not, as initially assumed, purely positive training through mask use. This often increases the subliminal drop in oxygen saturation SpO_2_ in the blood [23,28,29,30,32], which is already reduced by increased dead space volume and increased breathing resistance [18,31].

The overall possible resulting measurable drop in oxygen saturation O_2_ of the blood on the one hand [18,23,28,29,30,32] and the increase in carbon dioxide (CO_2_) on the other [13,15,19,21,22,23,24,25,26,27,28] contribute to an increased noradrenergic stress response, with heart rate increase [29,30,35] and respiratory rate increase [15,21,23,34], in some cases also to a significant blood pressure increase [25,35].

In panic-prone individuals, stress-inducing noradrenergic sympathetic activation can be partly directly mediated via the carbon dioxide (CO_2_) mechanism at the locus coeruleus in the brainstem [39,78,79,153], but also in the usual way via chemo-sensitive neurons of the nucleus solitarius in the medulla [136,154]. The nucleus solitarius [136] is located in the deepest part of the brainstem, a gateway to neuronal respiratory and circulatory control [154]. A decreased oxygen (O_2_) blood level there causes the activation of the sympathetic axis via chemoreceptors in the carotids [155,156].

Even subthreshold changes in blood gases such as those provoked when wearing a mask cause reactions in these control centers in the central nervous system. Masks, therefore, trigger direct reactions in important control centers of the affected brain via the slightest changes in oxygen and carbon dioxide in the blood of the wearer [136,154,155].

A link between disturbed breathing and cardiorespiratory diseases such as hypertension, sleep apnea and metabolic syndrome has been scientifically proven [56,57]. Interestingly, decreased oxygen/O_2_ blood levels and also increased carbon dioxide/CO_2_ blood levels are considered the main triggers for the sympathetic stress response [38,136]. The aforementioned chemo-sensitive neurons of the nucleus solitarius in the medulla are considered to be the main responsible control centers [136,154,155]. Clinical effects of prolonged mask-wearing would, thus, be a conceivable intensification of chronic stress reactions and negative influences on the metabolism leading towards a metabolic syndrome. The mask studies we found show that such disease-relevant respiratory gas changes (O_2_ and CO_2_) [38,136] are already achieved by wearing a mask [13,15,18,19,21,22,23,24,25,26,27,28,29,30,31,32,33,34].

A connection between hypoxia, sympathetic reactions and leptin release is scientifically known [136].

Additionally important is the connection of breathing with the influence on other bodily functions [56,57], including the psyche with the generation of positive emotions and drive [153]. The latest findings from neuro-psychobiological research indicate that respiration is not only a function regulated by physical variables to control them (feedback mechanism), but rather independently influences higher-level brain centers and, thus, also helps to shape psychological and other bodily functions and reactions [153,157,158]. Since masks impede the wearer’s breathing and accelerate it, they work completely against the principles of health-promoting breathing [56,57] used in holistic medicine and yoga. According to recent research, undisturbed breathing is essential for happiness and healthy drive [157,159], but masks work against this.

The result of significant changes in blood gases in the direction of hypoxia (drop in oxygen saturation) and hypercapnia (increase in carbon dioxide concentration) through masks, thus, has the potential to have a clinically relevant influence on the human organism even without exceeding normal limits.

According to the latest scientific findings, blood-gas shifts towards hypoxia and hypercapnia not only have an influence on the described immediate, psychological and physiological reactions on a macroscopic and microscopic level, but additionally on gene expression and metabolism on a molecular cellular level in many different body cells. Through this, the drastic disruptive intervention of masks in the physiology of the body also becomes clear down to the cellular level, e.g., in the activation of hypoxia-induced factor (HIF) through both hypercapnia and hypoxia-like effects [160]. HIF is a transcription factor that regulates cellular oxygen supply and activates signaling pathways relevant to adaptive responses. e.g., HIF inhibits stem cells, promotes tumor cell growth and inflammatory processes [160]. Based on the hypoxia- and hypercapnia-promoting effects of masks, which have been comprehensively described for the first time in our study, potential disruptive influences down to the intracellular level (HIF-a) can be assumed, especially through the prolonged and excessive use of masks. Thus, in addition to the vegetative chronic stress reaction in mask wearers, which is channeled via brain centers, there is also likely to be an adverse influence on metabolism at the cellular level. With the prospect of continued mask use in everyday life, this also opens up an interesting field of research for the future.

The fact that prolonged exposure to latently elevated CO_2_ levels and unfavorable breathing air compositions has disease-promoting effects was recognized early on. As early as 1983, the WHO described “Sick Building Syndrome” (SBS) as a condition in which people living indoors experienced acute disease-relevant effects that increased with time of their stay, without specific causes or diseases [161,162]. The syndrome affects people who spend most of their time indoors, often with subliminally elevated CO_2_ levels, and are prone to symptoms such as increased heart rate, rise in blood pressure, headaches, fatigue and difficulty concentrating [38,162]. Some of the complaints described in the mask studies we found (Figure 2) are surprisingly similar to those of Sick Building Syndrome [161]. Temperature, carbon dioxide content of the air, headaches, dizziness, drowsiness and itching also play a role in Sick Building Syndrome. On the one hand, masks could themselves be responsible for effects such as those described for Sick Building Syndrome when used for a longer period of time. On the other hand, they could additionally intensify these effects when worn in air-conditioned buildings, especially when masks are mandatory indoors. Nevertheless, there was a tendency towards higher systolic blood pressure values in mask wearers in some studies [21,31,34], but statistical significance was only found in two studies [25,35]. However, we found more relevant and significant evidence of heart rate increase, headache, fatigue and concentration problems associated with mask wearers (Figure 2) indicating the clinical relevance of wearing masks.

According to the scientific results and findings, masks have measurably harmful effects not only on healthy people, but also on sick people and their relevance is likely to increase with the duration of use [69]. Further research is needed here to shed light on the long-term consequences of widespread mask use with subthreshold hypoxia and hypercapnia in the general population, also regarding possible exacerbating effects on cardiorespiratory lifestyle diseases such as hypertension, sleep apnea and metabolic syndrome. The already often elevated blood carbon dioxide (CO_2_) levels in overweight people, sleep apnea patients and patients with overlap-COPD could possibly increase even further with everyday masks. Not only a high body mass index (BMI) but also sleep apnea are associated with hypercapnia during the day in these patients (even without masks) [19,163]. For such patients, hypercapnia means an increase in the risk of serious diseases with increased morbidity, which could then be further increased by excessive mask use [18,38].

The hypercapnia-induced effects of sympathetic stress activation are even cycle phase-dependent in women. Controlled by a progesterone mechanism, the sympathetic reaction, measured by increased blood pressure in the luteal phase, is considerably stronger [164]. This may also result in different sensitivities for healthy and sick women to undesirable effects masks have, which are related to an increase in carbon dioxide (CO_2_).

In our review, negative physical and psychological changes caused by masks could be objectified even in younger and healthy individuals.

The physical and chemical parameters did not exceed the normal values in most cases but were statistically significantly measurable (*p* < 0.05) tending towards pathological ranges. They were accompanied by physical impairments (see Figure 2). It is well known that subthreshold stimuli are capable of causing pathological changes when exposed to them for a long time: not only a single high dose of a disturbance, but also a chronically persistent, subthreshold exposure to it often leads to illness [38,46,47,48,50,51,52,53,54]. The scientifically repeatedly measurable physical and chemical mask effects were often accompanied by typical subjective complaints and pathophysiological phenomena. The fact that these frequently occur simultaneously and together indicates a syndrome under masks.

Figure 2 sums up the significant mask-dependent physiological, psychological, somatic and general pathological changes and their frequent occurrence together is striking. Within the framework of the quantitative evaluation of the experimental studies, we were actually able to prove a statistically significant correlation of the observed side effects of fatigue and oxygen depletion under mask use with *p* < 0.05. In addition, we found a frequent, simultaneous and joint occurrence of further undesirable effects in the scientific studies (Figure 2). Statistically significant associations of such co-occurring, adverse effects have already been described in primary studies [21,29]. We detected a combined occurrence of the physical parameter temperature rise under the mask with the symptom respiratory impairment in seven of the nine studies concerned (88%). We found a similar result for the decrease in oxygen saturation under mask and the symptom respiratory impairment with a simultaneous detection in six of the eight studies concerned (67%). We detected a combined occurrence of carbon dioxide rise under N95 mask use in nine of the 11 scientific papers (82%). We found a similar result for oxygen drop under N95 mask use with simultaneous co-occurrence in eight of 11 primary papers (72%). The use of N95 masks was also associated with headache in six of the 10 primary studies concerned (60%). A combined occurrence of the physical parameters temperature rise and humidity under masks was even found 100% within six of the six studies with significant measurements of these parameters (Figure 2).

**Figure 4:**
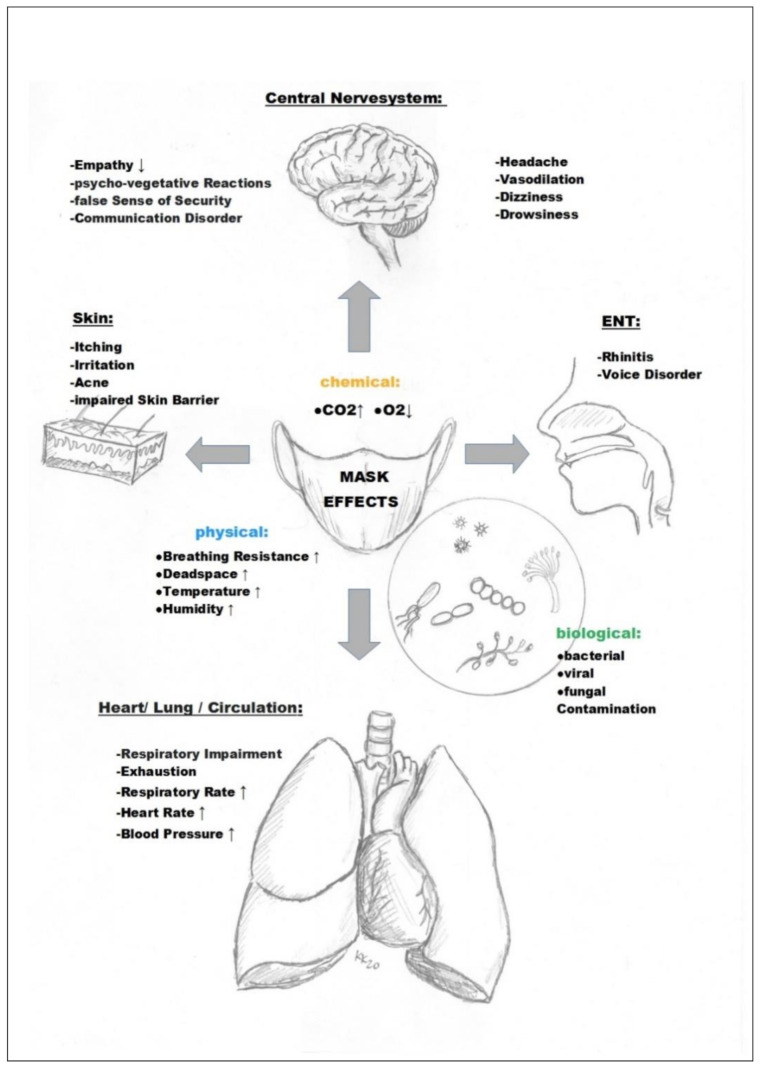
Unfavorable mask effects as components of Mask-Induced Exhaustion Syndrome (MIES). The chemical, physical and biological effects, as well as the organ system consequences mentioned, are all documented with statistically significant results in the scientific literature found (Figure 2). The term drowsiness is used here to summarize any qualitative neurological deficits described in the examined scientific literature.

Since the symptoms were described in combination in mask wearers and were not observed in isolation in the majority of cases, we refer to them as general Mask-Induced Exhaustion Syndrome (MIES) because of the consistent presentation in numerous papers from different disciplines. These include the following, predominantly statistically significantly (*p* < 0.05) proven pathophysiological changes and subjective complaints, which often occur in combination as described above (see also Section 3.1 to Section 3.11, Figure 2, Figure 3 and Figure 4):

- Increase in dead space volume [22,24,58,59] (Figure 3, Section 3.1 and Section 3.2).
- Increase in breathing resistance [31,35,61,118] (Figure 3, Figure 2: Column 8).
- Increase in blood carbon dioxide [13,15,19,21,22,23,24,25,26,27,28] (Figure 2: Column 5).
- Decrease in blood oxygen saturation [18,19,21,23,28,29,30,31,32,33,34] (Figure 2: Column 4).
- Increase in heart rate [15,19,23,29,30,35] (Figure 2: Column 12).
- Decrease in cardiopulmonary capacity [31] (Section 3.2).
- Feeling of exhaustion [15,19,21,29,31,32,33,34,35,69] (Figure 2: Column 14).
- Increase in respiratory rate [15,21,23,34] (Figure 2: Column 9).
- Difficulty breathing and shortness of breath [15,19,21,23,25,29,31,34,35,71,85,101,133] (Figure 2: Column 13).
- Headache [19,27,37,66,67,68,83] (Figure 2: Column 17).
- Dizziness [23,29] (Figure 2: Column 16).
- Feeling of dampness and heat [15,16,22,29,31,35,85,133] (Figure 2: Column 7).
- Drowsiness (qualitative neurological deficits) [19,29,32,36,37] (Figure 2: Column 15).
- Decrease in empathy perception [99] (Figure 2: Column 19).
- Impaired skin barrier function with acne, itching and skin lesions [37,72,73] (Figure 2: Column 20–22).

It can be deduced from the results that the effects described in healthy people are all more pronounced in sick people, since their compensatory mechanisms, depending on the severity of the illness, are reduced or even exhausted. Some existing studies on and with patients with measurable pathological effects of the masks support this assumption [19,23,25,34]. In most scientific studies, the exposure time to masks in the context of the measurements/investigations was significantly less (in relation to the total wearing and duration of use) than is expected of the general public under the current pandemic regulations and ordinances.

The exposure time limits are little observed or knowingly disregarded in many areas today as already mentioned in Section 3.11 on occupational medicine. The above facts allow the conclusion that the described negative effects of masks, especially in some of our patients and the very elderly, may well be more severe and adverse with prolonged use than presented in some mask studies.

From a doctor’s viewpoint, it may also be difficult to advise children and adults who, due to social pressure (to wear a mask) and the desire to feel they belong, suppress their own needs and concerns until the effects of masks have a noticeable negative impact on their health [76]. Nevertheless, the use of masks should be stopped immediately at the latest when shortness of breath, dizziness or vertigo occur [23,25]. From this aspect, it seems sensible for decision makers and authorities to provide information, to define instruction obligations and offer appropriate training for employers, teachers and other persons who have a supervisory or caregiving duty. Knowledge about first aid measures could also be refreshed and expanded accordingly in this regard.

Elderly, high-risk patients with lung disease, cardiac patients, pregnant women or stroke patients are advised to consult a physician to discuss the safety of an N95 mask as their lung volume or cardiopulmonary performance may be reduced [23]. A correlation between age and the occurrence of the aforementioned symptoms while wearing a mask has been statistically proven [19]. Patients with reduced cardiopulmonary function are at increased risk of developing serious respiratory failure with mask use according to the referenced literature [34]. Without the possibility of continuous medical monitoring, it can be concluded that they should not wear masks without close monitoring. The American Asthma and Allergy Society has already advised caution in the use of masks with regard to the COVID-19 pandemic for people with moderate and severe lung disease [165]. Since the severely overweight, sleep apnea patients and overlap-COPD sufferers are known to be prone to hypercapnia, they also represent a risk group for serious adverse health effects under extensive mask use [163]. This is because the potential of masks to produce additional CO_2_ retention may not only have a disruptive effect on the blood gases and respiratory physiology of sufferers, but may also lead to further serious adverse health effects in the long term. Interestingly, in an animal experiment an increase in CO_2_ with hypercapnia leads to contraction of smooth airway muscles with constriction of bronchi [166]. This effect could explain the observed pulmonary decompensations of patients with lung disease under masks (Section 3.2) [23,34].

Patients with renal insufficiency requiring dialysis are, according to the literature available, further candidates for a possible exemption from the mask requirement [34]. According to the criteria of the Centers for Disease Control and Prevention, GA, USA (CDC), sick and helpless people who cannot remove a mask on their own should be exempted from the mask requirement [82].

Since it can be assumed that children react even more sensitively to masks, the literature suggests that masks are a contraindication for children with epilepsies (hyperventilation as a trigger for seizures) [63]. In the field of pediatrics, special attention should also be paid to the mask symptoms described under psychological, psychiatric and sociological effects with possible triggering of panic attacks by CO_2_ rebreathing in the case of predisposition and also reinforcement of claustrophobic fears [77,78,79,167]. The mask-related disturbance of verbal [43,45,71] and non-verbal communication and, thus, of social interaction is particularly serious for children. Masks restrict social interaction and block positive perceptions (smiling and laughing) and emotional mimicry [42]. The proven mask-induced mild to moderate cognitive impairment with impaired thinking, decreased attention and dizziness [19,23,29,32,36,37,39,40,41,69], as well as the psychological and neurological effects [135], should be additionally taken into account when masks are compulsory at school and in the vicinity of both public and non-public transport, also regarding the possibility of an increased risk of accidents (see also occupational health side effects and hazards) [19,29,32,36,37]. The exclusion criteria mentioned in pediatric studies on masks (see pediatric impairments, Section 3.14) [26,133] should also apply to an exclusion of these children from the general mask obligation in accordance with the scientific findings for the protection of the sick children concerned. The long-term sociological, psychological and educational consequences of a comprehensive masking requirement extended to schools are also unpredictable with regard to the psychological and physical development of healthy children [42,135]. Interestingly, according to the Corona Thesis Paper of the University of Bremen children “are infected less often, they become ill less often, the lethality is close to zero, and they also pass on the infection less often”, according to the Thesis Paper 2.0 of the German University of Bremen on page 6 [138]. Studies conducted under real-life conditions with outcome endpoints showing hardly any infections, hardly any morbidity, hardly any mortality and only low contagiousness in children are clearly in the majority, according to Thesis Paper 3.0 of the German University of Bremen [138]. A recent German observational study (5600 reporting pediatricians) also showed a surprisingly low incidence of COVID-19 disease in children [168]. The infection of adults with SARS-CoV-2 by children has been considered in only one suspected case, but could not be proven with certainty, since the parents also had numerous contacts and exposure factors for viral infections due to their occupation. In this case, the circulating headlines in the public media that children contribute more to the incidence of infection are to be regarded as anecdotal.

In pregnant women, the use of masks during exertion or at rest over long periods of time is to be regarded as critical as little research has been done on this [20]. If there is clear scientific evidence of increased dead space ventilation with possible accumulation of CO_2_ in the mother’s blood, the use of masks by pregnant women for more than 1 h, as well as under physical stress, should be avoided in order to protect the unborn child [20,22]. The hypercapnia-promoting masks could act as a confounder of the fetal/maternal CO_2_ gradient in this case (Section 3.6) [20,22,28].

According to the literature cited in the Section 3.5 on psychiatric side effects (personality disorders with anxiety and panic attacks, claustrophobia, dementia and schizophrenia), masking should only be done, if at all, with careful consideration of the advantages and disadvantages. Attention should be paid to possible provocation of the number and severity of panic attacks [77,78,79].

In patients with headaches, a worsening of symptoms can be expected with prolonged mask use (see also Section 3.3., neurological side effects) [27,66,67,68]. As a result of the increase in blood carbon dioxide (CO_2_) when the mask is used, vasodilatation occurs in the central nervous system and the pulsation of the blood vessels decreases [27]. In this connection, it is also interesting to note radiological experiments that demonstrate an increase in brain volume under subthreshold, but still within normal limits of CO_2_ increase in the blood by means of structural MRI. The blood carbon dioxide increase was produced in seven subjects via rebreathing with resulting median carbon dioxide concentration of 42 mmHg and an interquartile range of 39.44 mmHg, corresponding to only a subthreshold increase given the normal values of 32–45 mmHg. In the experiment, there was a significant increase in brain parenchymal volume measurable under increased arterial CO_2_ levels (*p* < 0.02), with a concomitant decrease in CSF spaces (*p* < 0.04), entirely in accordance with the Monroe–Kelly doctrine, according to which the total volume within the skull always remains the same. The authors interpreted the increase in brain volume as an expression of an increase in blood volume due to a CO_2_ increase-induced dilation of the cerebral vessels [169]. The consequences of such equally subthreshold carbon dioxide (CO_2_) increases even under masks [13,15,18,19,22,23,25] are unclear for people with pathological changes inside the skull (aneurysms, tumors, etc.) with associated vascular changes [27] and brain volume shifts [169] especially due to longer exposure while wearing a mask, but could be of great relevance due to the blood gas-related volume shifts that take place.

In view of the increased dead space volume, the long-term and increased accumulation and rebreathing of other respiratory air components apart from CO_2_ is also unexplained, both in children and in old and sick people. Exhaled air contains over 250 substances, including irritant or toxic gases such as nitrogen oxides (NO), hydrogen sulfide (H2S), isoprene and acetone [170]. For nitrogen oxides [47] and hydrogen sulfide [46], pathological effects relevant to disease have been described in environmental medicine even at a low but chronic exposure [46,47,48]. Among the volatile organic compounds in exhaled air, acetone and isoprene dominate in terms of quantity, but allyl methyl sulfide, propionic acid and ethanol (some of bacterial origin) should also be mentioned [171]. Whether such substances also react chemically with each other underneath masks and in the dead space volume created by masks (Figure 3), and with the mask tissue itself, and in what quantities these and possible reaction products are rebreathed, has not yet been clarified. In addition to the blood gas changes described above (O_2_ drop and CO_2_ rise), these effects could also play a role with regard to undesirable mask effects. Further research is needed here and is of particular interest in the case of prolonged and ubiquitous use of masks.

The WHO sees the integration of individual companies and communities that produce their own fabric masks as a potential social and economic benefit. Due to the global shortage of surgical masks and personal protective equipment, it sees this as a source of income and points out that the reuse of fabric masks can reduce costs and waste and contribute to sustainability [2]. In addition to the question of certification procedures for such fabric masks, it should also be mentioned that due to the extensive mask obligation, textile (artificial) substances in the form of micro- and nanoparticles, some of which cannot be degraded in the body, are chronically absorbed into the body through inhalation to an unusual extent. In the case of medical masks, disposable polymers such as polypropylene, polyurethane, polyacrylonitrile, polystyrene, polycarbonate, polyethylene and polyester should be mentioned [140]. ENT physicians have already been able to detect such particles in the nasal mucosa of mask wearers with mucosal reactions in the sense of a foreign body reaction with rhinitis [96]. In the case of community masks, other substances from the textile industry are likely to be added to those mentioned above. The body will try to absorb these substances through macrophages and scavenger cells in the respiratory tract and alveoli as part of a foreign body reaction, whereby toxin release and corresponding local and generalized reactions may occur in an unsuccessful attempt to break them down [172]. Extensive respiratory protection in permanent long-term use (24/7), at least from a theoretical point of view, also potentially carries the risk of leading to a mask-related pulmonary [47] or even generalized disorder, as is already known from textile workers chronically exposed to organic dusts in the Third World (byssinosis) [172].

For the general public, from a scientific angle, it is necessary to draw on the long-standing knowledge of respiratory protection in occupational medicine in order to protect children in particular from harm caused by uncertified masks and improper use.

The universal undefined and extended mask requirement—without taking into account multiple predispositions and susceptibilities—contradicts the claim of an increasingly important individualized medicine with a focus on the unique characteristics of each individual [173].

A systematic review on the topic of masks is necessary according to the results of our scoping review. The primary studies often showed weaknesses in operationalization, especially in the evaluation of cognitive and neuropsychological parameters. Computerized test procedures will be useful here in the future. Mask research should also set itself the future goal of investigating and defining subgroups for whom respiratory protection use is particularly risky.

## 5. Limitations

Our approach with a focus on negative effects is in line with Villalonga-Olives and Kawachi [12]. With the help of such selective questioning in the sense of dialectics, new insights can be gained that might otherwise have remained hidden. Our literature search focused on adverse negative effects of masks, in particular to point out risks especially for certain patient groups. Therefore, publications presenting only positive effects of masks were not considered in this review.

For a compilation of studies with harmless results when using masks, reference must, therefore, be made to reviews with a different research objective, whereby attention must be paid to possible conflicts of interest there. Some of the studies excluded by us lacking negative effects have shown methodological weaknesses (small, non-uniform experimental groups, missing control group even without masks due to corona constraints, etc.) [174]. In other words, if no negative concomitant effects were described in publications, it does not necessarily mean that masks have exclusively positive effects. It is quite possible that negative effects were simply not mentioned in the literature and the number of negative effects may well be higher than our review suggests.

We only searched one database, so the number of papers on negative mask effects may be higher than we reported.

In order to be able to describe characteristic effects for each mask type even more extensively, we did not have enough scientific data on the respective special designs of the masks. There is still a great need for research in this area due to the current pandemic situation with extensive mandatory masking.

In addition, the experiments evaluated in this paper do not always have uniform measurement parameters and study variables and, depending on the study, take into account the effect of masks at rest or under stress with subjects having different health conditions. Figure 2, therefore, represents a compromise. The results of the primary studies on mask use partially showed no natural variation in parameters, but often showed such clear correlations between symptoms and physiological changes, so that a statistical correlation analysis was not always necessary. We found a statistically significant correlation of oxygen deprivation and fatigue in 58% of the studies (*p* < 0.05). A statistically significant correlation evidence for other parameters has been previously demonstrated in primary studies [21,29].

The most commonly used personal particulate matter protective equipment in the COVID-19 pandemic is the N95 mask [23]. Due to its characteristics (better filtering function, but greater airway resistance and more dead space volume than other masks), the N95 mask is able to highlight negative effects of such protective equipment more clearly than others (Figure 3). Therefore, a relatively frequent consideration and evaluation of N95 masks within the studies found (30 of the 44 quantitatively evaluated studies, 68%) is even advantageous within the framework of our research question. Nevertheless, it remains to be noted that the community masks sold on the market are increasingly similar to the protective equipment that has been better investigated in scientific studies, such as surgical masks and N95 masks, since numerous manufacturers and users of community masks are striving to approximate the professional standard (surgical mask, N95/FFP2). Recent study results on community masks indicate similar effects for respiratory physiology as described for medical masks: in a recent publication, fabric masks (community masks) also provoked a measurable increase in carbon dioxide PtcCO_2_ in wearers during exertion and came very close to surgical masks in this effect [21].

Most of the studies cited in our paper included only short observation and application periods (mask-wearing durations investigated ranged from 5 min [26] to 12 h [19]. In only one study, a maximum observation period of an estimated 2-month period was chosen [37]. Therefore, the actual negative effects of masks over a longer application period might be more pronounced than presented in our work.

## 6. Conclusions

On the one hand, the advocacy of an extended mask requirement remains predominantly theoretical and can only be sustained with individual case reports, plausibility arguments based on model calculations and promising in vitro laboratory tests. Moreover, recent studies on SARS-CoV-2 show both a significantly lower infectivity [175] and a significantly lower case mortality than previously assumed, as it could be calculated that the median corrected infection fatality rate (IFR) was 0.10% in locations with a lower than average global COVID-19 population mortality rate [176]. In early October 2020, the WHO also publicly announced that projections show COVID-19 to be fatal for approximately 0.14% of those who become ill—compared to 0.10% for endemic influenza—again a figure far lower than expected [177].

On the other hand, the side effects of masks are clinically relevant.

In our work, we focused exclusively on the undesirable and negative side effects that can be produced by masks. Valid significant evidence of combined mask-related changes were objectified (*p* < 0.05, *n* ≥ 50%), and we found a clustered and common occurrence of the different adverse effects within the respective studies with significantly measured effects (Figure 2). We were able to demonstrate a statistically significant correlation of the observed adverse effect of hypoxia and the symptom of fatigue with *p* < 0.05 in the quantitative evaluation of the primary studies. Our review of the literature shows that both healthy and sick people can experience Mask-Induced Exhaustion Syndrome (MIES), with typical changes and symptoms that are often observed in combination, such as an increase in breathing dead space volume [22,24,58,59], increase in breathing resistance [31,35,60,61], increase in blood carbon dioxide [13,15,17,19,21,22,23,24,25,26,27,28,29,30,35], decrease in blood oxygen saturation [18,19,21,23,28,29,30,31,32,33,34], increase in heart rate [23,29,30,35], increase in blood pressure [25,35], decrease in cardiopulmonary capacity [31], increase in respiratory rate [15,21,23,34,36], shortness of breath and difficulty breathing [15,17,19,21,23,25,29,31,34,35,60,71,85,101,133], headache [19,27,29,37,66,67,68,71,83], dizziness [23,29], feeling hot and clammy [17,22,29,31,35,44,71,85,133], decreased ability to concentrate [29], decreased ability to think [36,37], drowsiness [19,29,32,36,37], decrease in empathy perception [99], impaired skin barrier function [37,72,73] with itching [31,35,67,71,72,73,91,92,93], acne, skin lesions and irritation [37,72,73], overall perceived fatigue and exhaustion [15,19,21,29,31,32,34,35,69] (Figure 2, Figure 3 and Figure 4).

Wearing masks does not consistently cause clinical deviations from the norm of physiological parameters, but according to the scientific literature, a long-term pathological consequence with clinical relevance is to be expected owing to a longer-lasting effect with a subliminal impact and significant shift in the pathological direction. For changes that do not exceed normal values, but are persistently recurring, such as an increase in blood carbon dioxide [38,160], an increase in heart rate [55] or an increase in respiratory rate [56,57], which have been documented while wearing a mask [13,15,17,19,21,22,23,24,25,26,27,28,29,30,34,35] (Figure 2), a long-term generation of high blood pressure [25,35], arteriosclerosis and coronary heart disease and of neurological diseases is scientifically obvious [38,55,56,57,160]. This pathogenetic damage principle with a chronic low-dose exposure with long-term effect, which leads to disease or disease-relevant conditions, has already been extensively studied and described in many areas of environmental medicine [38,46,47,48,49,50,51,52,53,54]. Extended mask-wearing would have the potential, according to the facts and correlations we have found, to cause a chronic sympathetic stress response induced by blood gas modifications and controlled by brain centers. This in turn induces and triggers immune suppression and metabolic syndrome with cardiovascular and neurological diseases.

We not only found evidence in the reviewed mask literature of potential long-term effects, but also evidence of an increase in direct short-term effects with increased mask-wearing time in terms of cumulative effects for: carbon dioxide retention, drowsiness, headache, feeling of exhaustion, skin irritation (redness, itching) and microbiological contamination (germ colonization) [19,22,37,66,68,69,89,91,92].

Overall, the exact frequency of the described symptom constellation MIES in the mask-using populace remains unclear and cannot be estimated due to insufficient data.

Theoretically, the mask-induced effects of the drop in blood gas oxygen and increase in carbon dioxide extend to the cellular level with induction of the transcription factor HIF (hypoxia-induced factor) and increased inflammatory and cancer-promoting effects [160] and can, thus, also have a negative influence on pre-existing clinical pictures.

In any case, the MIES potentially triggered by masks (Figure 3 and Figure 4) contrasts with the WHO definition of health: “health is a state of complete physical, mental and social well-being and not merely the absence of disease or infirmity.” [178].

**Figure 5:**
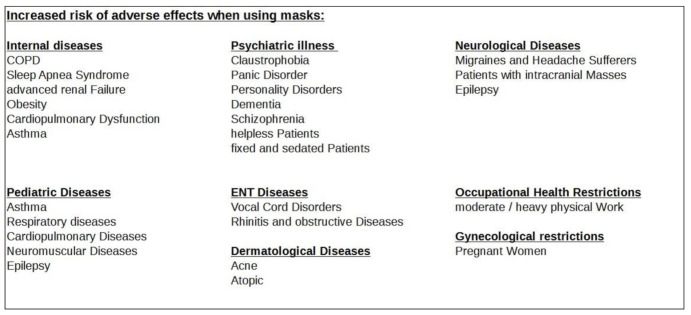
Diseases/predispositions with significant risks, according to the literature found, when using masks. Indications for weighing up medical mask exemption certificates.

All the scientific facts found in our work expand the knowledge base for a differentiated view of the mask debate. This gain can be relevant for decision makers who have to deal with the issue of mandatory mask use during the pandemic under constant review of proportionality as well as for physicians who can advise their patients more appropriately on this basis. For certain diseases, taking into account the literature found in this study, it is also necessary for the attending physician to weigh up the benefits and risks with regard to a mask obligation. With an overall strictly scientific consideration, a recommendation for mask exemption can become justifiable within the framework of a medical appraisal (Figure 5).

In addition to protecting the health of their patients, doctors should also base their actions on the guiding principle of the 1948 Geneva Declaration, as revised in 2017. According to this, every doctor vows to put the health and dignity of his patient first and, even under threat, not to use his medical knowledge to violate human rights and civil liberties [9]. Within the framework of these findings, we, therefore, propagate an explicitly medically judicious, legally compliant action in consideration of scientific factual reality [2,4,5,16,130,132,143,175,176,177] against a predominantly assumption-led claim to a general effectiveness of masks, always taking into account possible unwanted individual effects for the patient and mask wearer concerned, entirely in accordance with the principles of evidence-based medicine and the ethical guidelines of a physician.

The results of the present literature review could help to include mask-wearing in the differential diagnostic pathophysiological cause consideration of every physician when corresponding symptoms are present (MIES, Figure 4). In this way, the physician can draw on an initial complaints catalogue that may be associated with mask-wearing (Figure 2) and also exclude certain diseases from the general mask requirement (Figure 5).

For scientists, the prospect of continued mask use in everyday life suggests areas for further research. In our view, further research is particularly desirable in the gynecological (fetal and embryonic) and pediatric fields, as children are a vulnerable group that would face the longest and, thus, most profound consequences of a potentially risky mask use. Basic research at the cellular level regarding mask-induced triggering of the transcription factor HIF with potential promotion of immunosuppression and carcinogenicity also appears to be useful under this circumstance. Our scoping review shows the need for a systematic review.

The described mask-related changes in respiratory physiology can have an adverse effect on the wearer’s blood gases sub-clinically and in some cases also clinically manifest and, therefore, have a negative effect on the basis of all aerobic life, external and internal respiration, with an influence on a wide variety of organ systems and metabolic processes with physical, psychological and social consequences for the individual human being.

## Data Availability

Not applicable.
